# Characterizing glucokinase variant mechanisms using a multiplexed abundance assay

**DOI:** 10.1186/s13059-024-03238-2

**Published:** 2024-04-16

**Authors:** Sarah Gersing, Thea K. Schulze, Matteo Cagiada, Amelie Stein, Frederick P. Roth, Kresten Lindorff-Larsen, Rasmus Hartmann-Petersen

**Affiliations:** 1 The Linderstrøm-Lang Centre for Protein Science, Department of Biology, University of Copenhagen, Ole Maaløes Vej 5, DK-2200 Copenhagen, Denmark; 2 Donnelly Centre, University of Toronto, M5S 3E1 Toronto, ON Canada; 3 Department of Molecular Genetics, University of Toronto, M5S 1A8 Toronto, ON Canada; 4 Lunenfeld-Tanenbaum Research Institute, Sinai Health, M5G 1X5 Toronto, ON Canada; 5 Department of Computational and Systems Biology, University of Pittsburgh School of Medicine, 15213 Pittsburgh, USA

**Keywords:** MAVE, DMS, Protein stability, Protein dynamics, GCK

## Abstract

**Background:**

Amino acid substitutions can perturb protein activity in multiple ways. Understanding their mechanistic basis may pinpoint how residues contribute to protein function. Here, we characterize the mechanisms underlying variant effects in human glucokinase (GCK) variants, building on our previous comprehensive study on GCK variant activity.

**Results:**

Using a yeast growth-based assay, we score the abundance of 95% of GCK missense and nonsense variants. When combining the abundance scores with our previously determined activity scores, we find that 43% of hypoactive variants also decrease cellular protein abundance. The low-abundance variants are enriched in the large domain, while residues in the small domain are tolerant to mutations with respect to abundance. Instead, many variants in the small domain perturb GCK conformational dynamics which are essential for appropriate activity.

**Conclusions:**

In this study, we identify residues important for GCK metabolic stability and conformational dynamics. These residues could be targeted to modulate GCK activity, and thereby affect glucose homeostasis.

**Supplementary Information:**

The online version contains supplementary material available at 10.1186/s13059-024-03238-2.

## Background

Protein function is crucial for cellular and organismal homeostasis, but can be perturbed by missense variants through various mechanisms. For instance, amino acid substitutions in active site residues can directly affect protein activity, but in general such residues only constitute a small fraction of a protein. Conversely, many residues affect the thermodynamic folding stability of a protein. As most proteins need to fold into their native conformation to be functional, a widespread consequence of missense variants is decreased protein stability, leading to protein unfolding, degradation, and a decreased protein abundance in the cell [1–5]. In addition, variants may affect other functional sites than catalytic residues, such as interaction interfaces and allosteric sites. Missense variants may therefore result in the same phenotype through multiple independent mechanisms that may be hard to disambiguate [6, 7]. Understanding the molecular mechanisms of protein variants not only improves our general understanding of protein function but is also important for interpreting and interfering with the effects of disease-causing variants.

Pathogenic variants in the glucokinase gene (*GCK*) are linked to at least three diseases. Heterozygous variants that increase activity lead to hyperinsulinemic hypoglycemia (HH, MIM# 601820), where insulin is secreted at low blood glucose levels [8, 9]. Conversely, variants that decrease activity are linked to diabetes: GCK-maturity-onset diabetes of the young (GCK-MODY, MIM# 125851) when heterozygous [10, 11] and permanent neonatal diabetes mellitus (PNDM, MIM# 606176) if homozygous or compound heterozygous [12, 13]. These glucose homeostasis diseases arise due to improper insulin secretion, which in pancreatic $\beta$-cells is regulated by the rate of glucose phosphorylation, catalyzed by GCK [14–16].

GCK is a 465-residue monomeric protein that folds into a small and a large domain [17]. Between the two domains is the single active site where glucose binds and becomes phosphorylated to form glucose-6-phosphate. Binding of glucose to GCK modulates the enzyme’s conformational landscape which includes multiple stable conformations [18, 19]. In the absence of glucose, GCK primarily populates the inactive super-open state, characterized by a large opening angle between the two domains and intrinsical disorder of an active site loop (residues 150–179) [17]. Upon glucose binding, GCK shifts towards a more compact active state, known as the closed state [17]. Here, the distance between the two domains is reduced, the small domain is structurally re-organized, and the 150–179 loop folds into a $\beta$-hairpin, collectively resulting in a catalytically active conformation. The conformational dynamics between inactive and active states occur on a millisecond timescale that is comparable to $k_{cat}$ [20], which enables the dynamics to modulate GCK activity. Therefore, GCK has a sigmoidal response to glucose, which is essential for appropriate GCK activity [17, 21].

Previously, using functional complementation in yeast, we characterized the activity of 9003 out of 9280 possible (97%) GCK missense and nonsense variants [22]. Accordingly, we now know the functional impact of most variants. However, the mechanisms leading to altered enzyme activity remain largely unknown. Prior mechanistic analyses of a few hyperactive variants found that some altered the dynamics and/or structure of the 150–179 loop [23], while others lead to a more compact conformation in the absence of glucose, similar to the closed state [24–26]. Building on this, we found that a conformational shift towards the active state could be a widespread mechanism for hyperactive variants [22]. The mechanisms of hypoactive variants include reduced structural stability and cellular abundance [27, 28], which was found to be a major determinant of the phenotypic severity in PNDM patients [29]. In addition, a conformational shift towards the inactive state has been predicted to be the mechanism of five hypoactive variants using molecular dynamics (MD) simulations [30]. However, the mechanistic basis of especially hypoactive variants remains to be examined more broadly.

Here, we use a yeast-based growth assay to determine the abundance of 8822 (95%) GCK missense and nonsense variants. Abundance was decreased by amino acid substitutions in buried regions of the large domain. Conversely, in the small domain, variants in general had little effect on abundance and instead affected the conformational dynamics of GCK. Collectively, our results expand the knowledge on the mechanisms of GCK disease-causing variants and illuminate the interplay between protein dynamics and abundance in determining GCK function.

## Results and discussion

### Measuring glucokinase variant abundance using the DHFR-PCA

**Fig. 1:**
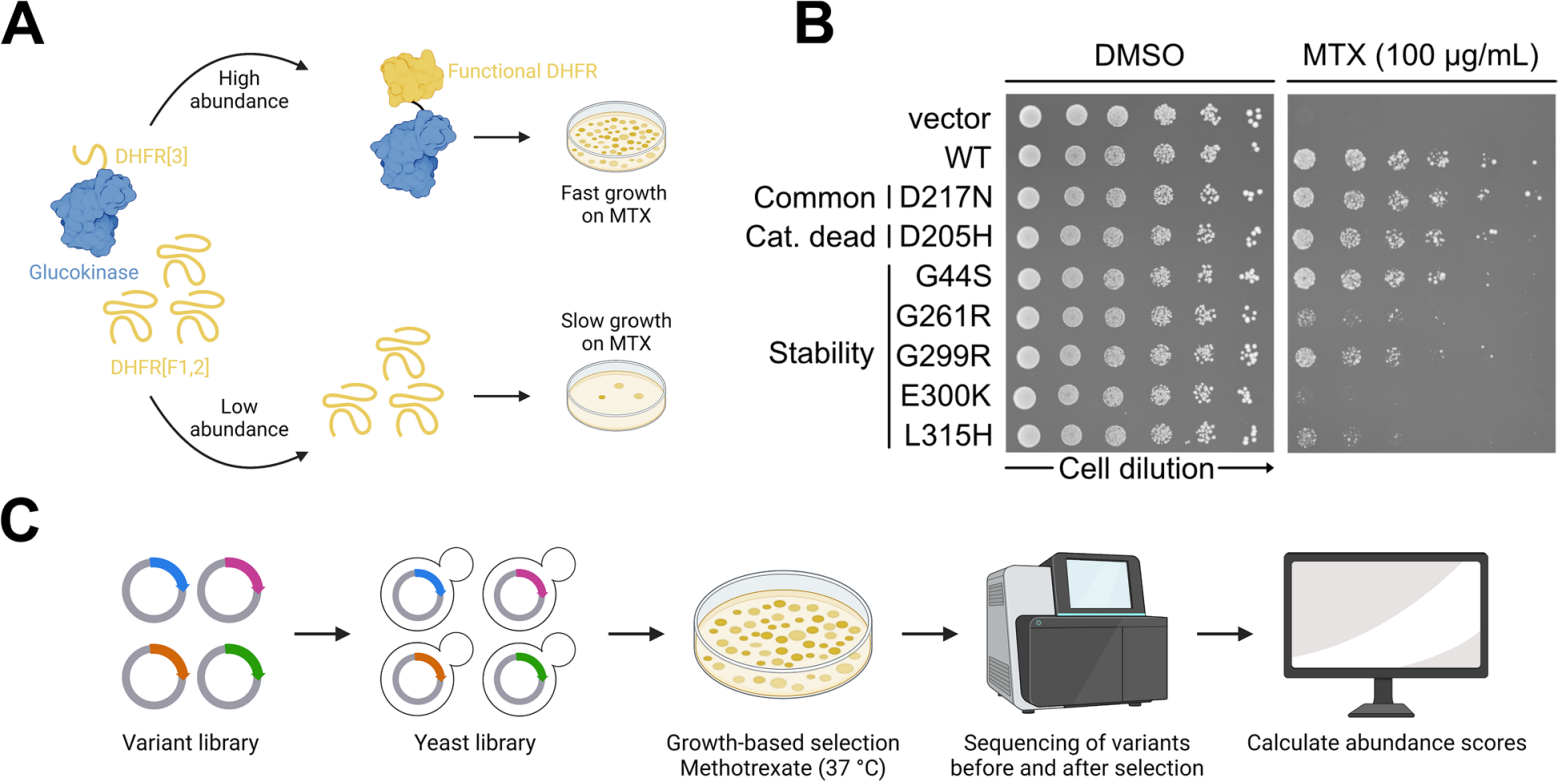
Measuring glucokinase variant abundance by DHFR-PCA. **A** Overview of the Dihydrofolate Reductase Protein-Fragment Complementation Assay (DHFR-PCA). **B** Low-throughput test of the DHFR-PCA. Selected glucokinase variants expressed in wild-type yeast cells were grown on medium without (DMSO) or with methotrexate (MTX) to assess their impact on cellular protein abundance. The vector control did not contain the DHFR-PCA sequences. **C** Overview of the multiplexed assay for glucokinase variant abundance

In order to assay GCK variant abundance, we used the Dihydrofolate Reductase Protein-Fragment Complementation Assay (DHFR-PCA) [31–34]. In this system, a methotrexate-resistant mutant of mouse DHFR is split into two fragments. One fragment (DHFR[F3]) is fused to the protein of interest, here GCK, while the other fragment (DHFR [F1,2]) is over-expressed freely. Both the fusion protein and fragment are expressed in wild-type yeast cells, which are grown on media containing methotrexate to inhibit the endogenous DHFR. If the fusion protein is abundant in the cell, the two DHFR fragments will reconstitute to form functional DHFR, thus enabling the cell to grow on methotrexate medium. However, if the fusion protein has a low abundance in the cell, less of the functional DHFR will form, leading to slower growth. In this way, yeast growth reports on protein abundance (Fig. 1A).

To test the dynamic range of the DHFR-PCA for GCK missense variants, we assayed the wild-type protein and seven selected variants in low-throughput. Yeast expressing wild-type GCK grew on methotrexate medium, while an empty vector control showed no growth (Fig. 1B). The common variant D217N [35] and a catalytically inactive variant D205H [17, 36] grew similar to wild-type GCK (Fig. 1B), suggesting a wild-type-like abundance as expected. The five remaining variants (G44S, G261R, G299R, E300K, and L315H) are disease-linked [37–39] and were previously predicted by thermodynamic stability calculations ($\Delta \Delta G$) to be destabilized [22]. In addition, E300K is a well-studied unstable GCK variant [27, 28]. Accordingly, all five variants showed reduced growth compared to wild-type GCK (Fig. 1B), albeit for the G44S and G299R variants this effect was less pronounced. In conclusion, the DHFR-PCA detected the low-abundance variants and can therefore assess GCK variant abundance.

Next, we multiplexed the DHFR-PCA to widely assess the abundance of GCK missense variants (Fig. 1C). Previously, we generated a library of GCK variants [22]. This library was cloned into the DHFR-PCA vector to generate an abundance variant library, which was transformed into wild-type yeast. Following outgrowth, the yeast library was grown at 37 ^∘^C on methotrexate medium for 4 days to select for abundance. Then, variants were sequenced before and after selection, and sequencing data were analyzed to obtain abundance scores.

### A map of glucokinase variant abundance

**Fig. 2:**
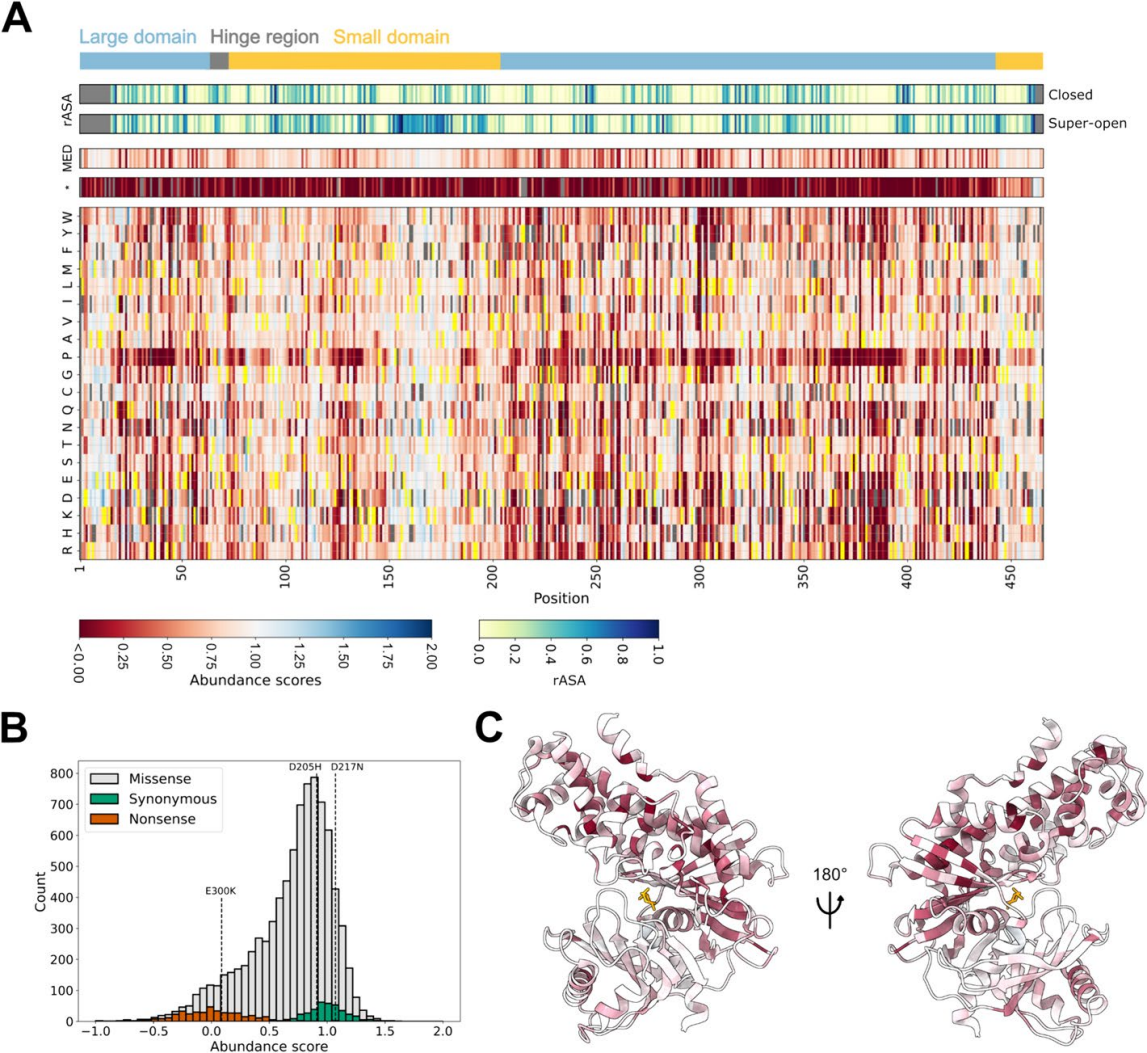
Map of glucokinase variant abundance. **A** Heatmap showing the abundance scores of 8822 missense and nonsense (*) glucokinase variants, as well as the median score at each position (MED). The wild-type amino acid at each position is shown in yellow. Missing variants are shown in gray. Above the heatmap, the regions forming the hinge region (gray) and the large (light blue) and small (light orange) domains are represented as a bar. In addition, the relative solvent accessible surface area (rASA) of each residue in the closed and super-open conformations is shown. **B** Abundance score distributions of glucokinase missense, synonymous, and nonsense variants. Dashed lines indicate the scores of three variants tested in low-throughput to be unstable (E300K) or wild-type-like (D205H and D217N). **C** The closed active state of glucokinase colored by median abundance scores. The coloring scheme is the same as in panel **A**. Glucose is shown in orange. PDB: 1V4S

We scored the abundance of 8822 missense and nonsense variants (95%) (Fig. 2A). The abundance scores are scaled such that nonsense variants have a median score of zero while synonymous variants have a median score of one. As an initial quality control check of the abundance scores, we found that the variants tested in low-throughput scored as expected (Additional file 1: Fig. S1A). In addition, the distributions of nonsense and synonymous variants were separated, while the scores of missense variants spanned from synonymous-like to nonsense-like (Fig. 2B). Consistent with expectations, nonsense mutations at most positions were not tolerated except for at the extreme C-terminal region (Fig. 2A). To further validate the abundance scores, we examined the cellular protein levels of 11 GCK variants expressed with an N-terminal GFP-tag, using western blotting. The protein levels quantified from western blots correlated with abundance scores (Pearson’s *r =* 0.80, *p*-value = 2e−08) (Additional file 1: Fig. S1BC). We note that variants with an abundance score below 0.5 all showed low cellular protein abundance and that differences in scores below 0.5 may not translate to changes in cellular protein levels. Despite this limitation, the abundance scores reflect cellular protein abundance of the GCK variants.

Having validated the abundance scores, we examined variant effects structurally, mapping the median abundance score at each position onto the structure of glucose-bound GCK. This revealed that the small domain tolerated mutations at most positions (Fig. 2C), potentially due to the domain’s conformational heterogeneity and dynamic nature [17, 24]. In contrast, the large domain is more static [17, 24], and while surface-exposed residues seemed mutation-tolerant, most buried residues in the large domain appeared to destabilize GCK when mutated (Fig. 2C, Additional file 1: Fig. S2). Accordingly, 33% of residues in the large domain had a low median abundance score (< 0.58, Additional file 1: Fig. S3), compared to 10% of small-domain residues (Additional file 1: Fig. S4). The ratios of low abundance residues to buried residues were 57% (55%) and 31% (17%) for the large and small domain, respectively, when evaluated on the super-open (closed) structure. Thus, low abundance variants were still enriched in the large domain when the difference in the number of buried residues in the two domains was taken into account. Hence, for loss-of-function variants in the large domain, a general mechanism may be to decrease protein abundance.

### Mechanistic analyses of hypo- and hyperactive glucokinase variants

**Fig. 3:**
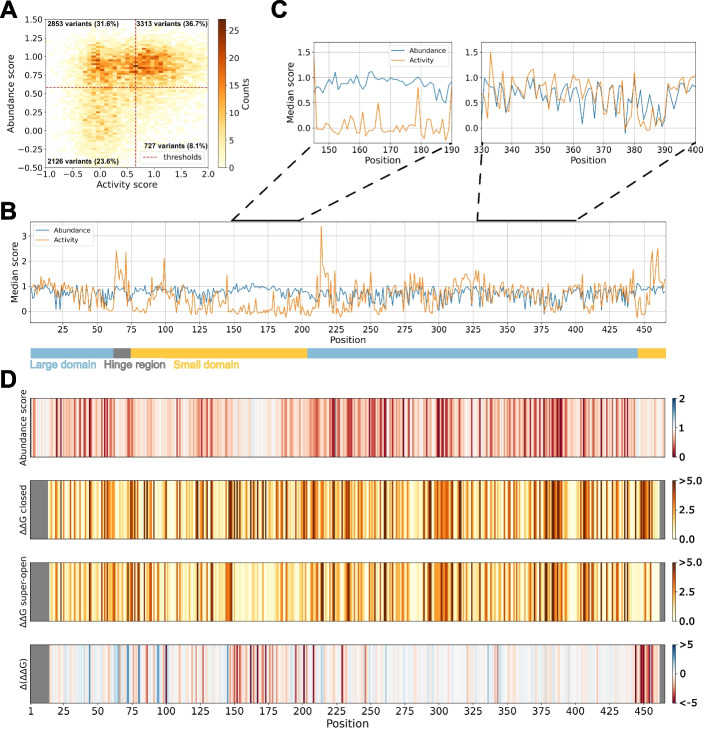
Changes in glucokinase activity explained by decreased abundance and conformational shifts. **A** Abundance and activity scores of 9019 missense, nonsense, and synonymous glucokinase variants shown as a 2D histogram. The thresholds for low abundance (0.58) and low activity (0.66) are indicated as red dashed lines. The number and percentage of variants falling within each quadrant are reported. **B** The median abundance and activity of variants at each position of the glucokinase sequence is shown as a line plot. The regions forming the hinge region (gray) and the large (light blue) and small (light orange) domains are represented as a bar at the bottom. **C** Plots zooming in on regions 145–190 (left) and 330–400 (right) from panel **B**. **D** Barcode plots showing the median abundance score, predicted change in protein thermodynamic stability ($\Delta \Delta G$, kcal/mol) using the closed active state or the super-open inactive state, and the difference $(\Delta (\Delta \Delta G))$ between the two $\Delta \Delta G$ predictions. For the bottom plot, red indicates that variants at these positions are predicted to destabilize the closed state more, while at blue positions variants are predicted to destabilize the super-open state more. PDBs: 1V4S (closed) and 1V4T (super-open). The $\Delta \Delta G$ data were obtained from [22] except for the 157–179 region in the super-open state. For all panels, the data on glucokinase variant activities were obtained from [22]

As decreased protein stability is a major cause of loss-of-function variants [1, 40], we next examined the relation between GCK variant abundance and activity. We used GCK variant activity scores previously determined using yeast complementation [22]. An activity score of zero corresponds to the activity of nonsense variants, while a score of one corresponds to the activity of synonymous variants. As expected, activity scores show a greater correlation with evolutionary distance scores (Additional file 1: Figs. S5 and S6) while abundance scores correlate with predictions of changes in protein thermodynamic stability ($\Delta \Delta G$, Additional file 1: Figs. S7 and S8). Using the activity and abundance scores of 9019 variants (including missense, synonymous, and nonsense variants), we examined how many hypoactive variants were associated with decreased abundance. We used a threshold of 0.58 for low abundance (Additional file 1: Fig. S3) and 0.66 for low activity [22]. Using these thresholds, 43% of the low-activity variants decreased abundance (Fig. 3A), in line with prior analyses [2, 7, 41] and similar to what is found using $\Delta \Delta G$ values with a threshold of 2 kcal/mol [41] (43% using the super-open structure and 53% using the closed structure). The fraction of variants with decreased activity showing low abundance was similar for pathogenic variants causing GCK-MODY [42] (41%, Additional file 1: Fig. S9), suggesting that loss of abundance is a prevalent mechanism for GCK disease-associated mutations. The remaining 57% low-activity variants were not associated with low abundance and appeared to lose activity through other mechanisms, including a predicted decreased ligand binding affinity (Additional file 1: Fig. S10).

Surprisingly, 25% of the low-abundance variants scored as wild-type-like or hyperactive in the activity assay (Fig. 3A). This apparent discrepancy might stem from different assay conditions, such as expression level or temperature (activity was assayed at 30 ^∘^C, while abundance was assayed at 37 ^∘^C), e.g., some low-abundance but active variants might be temperature-sensitive or compensated for by high expression. Alternatively, some variants might reduce abundance but increase specific activity, resulting in a wild-type-like or increased activity score, as the activity assay also to some extent reflects variant abundance. In conclusion, decreased abundance appears to be a major mechanism for GCK variants with decreased activity, in particular in the large domain, although the association between abundance and activity is not simple.

In order to identify the regions of GCK where changes in activity upon mutation are not explained by abundance, we compared the median activity and abundance scores along the GCK sequence (Fig. 3B). In some regions, the two medians showed a good correlation (Fig. 3B and C right panel), suggesting that loss-of-activity variants at these positions are caused by decreased abundance. In contrast, some regions showed large deviations between the two scores (Fig. 3B and C left panel). In general, regions with increased activity appeared unaffected in the abundance assay (Fig. 3B), suggesting that a changed abundance is not a common mechanism for hyperactive variants. Notably, nearly all regions where variants increased or decreased activity without affecting abundance are part of the small domain (Fig. 3B).

The small domain attains several conformations during GCK’s catalytic cycle [17, 24]. Consequently, small-domain variants might affect GCK activity by altering GCK dynamics. Such a mechanism is well-established for hyperactive variants [22–26]. For hypoactive variants, MD simulations have predicted five small-domain variants to shift GCK towards inactive conformations [30]. In addition, we previously used predictions of protein thermodynamic stability ($\Delta \Delta G$) for the structures of super-open and closed GCK to examine a conformational shift mechanism [22]. Although we mostly focused on hyperactive variants, we found two regions around residues 150 and 450 where hypoactive variants were predicted to shift GCK towards the inactive state. Accordingly, the region around residue 450, corresponding to helix 13, was previously found to modulate the allosteric properties of GCK [43].

Our prior mechanistic analysis of hypoactive variants was limited by residues 157–179 missing from the crystal structure of super-open GCK. To examine this region further, we created five different structural models of the super-open state that included the 157–179 loop region, assuming that the region is disordered, as previously seen for all prominent substates of unliganded GCK [20]. For all five models, we predicted the change in protein thermodynamic stability using Rosetta [44] and used the average $\Delta \Delta G$s from the five models for the missing loop residues to supplement our previous predictions [22]. As previously, we calculated the difference between the $\Delta \Delta G$s in the closed and super-open state ($\Delta \Delta G_\text {super-open}-\Delta \Delta G_\text {closed}$). Variants with a high negative score are predicted to shift GCK towards the inactive (super-open) state, given that they do not severely destabilize the super-open conformation, which would likely lead to decreased cellular abundance. Many residues were on average predicted to shift GCK towards the super-open conformation upon mutation, and these spanned the entire 150–179 region (Fig. 3D). Variants in the 150–179 region might therefore severely decrease activity without affecting abundance by shifting GCK into an inactive state.

### Variants in the 150–179 region affect glucokinase conformational dynamics

**Fig. 4:**
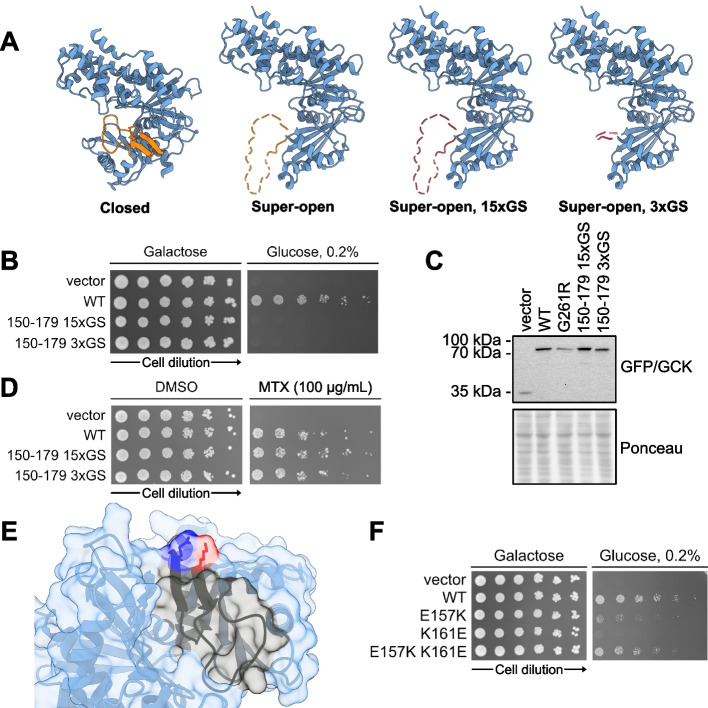
A conformational shift towards the super-open state as a mechanism for variants in the 150–179 region. **A** Left, protein structures of wild-type glucokinase in the closed and super-open states with the 150–179 region marked in orange. Right, overview of glucokinase in the super-open state with the 150–179 region substituted by 30 (15xGS) or 6 residues of GS (3xGS) shown in red. **B** Yeast growth assay scoring the activity of wild-type glucokinase (WT) and the two mutants. The growth on galactose is used as a control while growth on 0.2% glucose reflects glucokinase activity. **C** Western blot showing the protein levels of the indicated constructs expressed in the *hxk1*$\Delta$ *hxk2*$\Delta$ *glk1*$\Delta$ yeast strain from panel **B**. **D** DHFR-PCA probing the abundance of wild-type glucokinase (WT) and the two mutants by growing yeast cells on control medium (DMSO) and medium with methotrexate (MTX) to select for abundance. **E** Structure of glucokinase in the closed state with the 150–179 region marked in black, E157 in dark blue and K161 in red. **F** Yeast growth assay scoring the activity of wild-type glucokinase (WT) and the indicated single and double mutants. PDBs: 1V4S (closed) and 1V4T (super-open)

To substantiate a conformational shift mechanism for hypoactive variants experimentally, we focused on the 150–179 region. If the region’s disorder in the super-open state results in mutational tolerance with respect to abundance, then any disordered sequence should be tolerated without perturbing GCK protein abundance. To test this, we replaced the region spanning residues 150–179 with a GS-repeat sequence of either 30 or 6 residues (Fig. 4A). The resulting mutants retained no detectable activity (Fig. 4B), as expected, but did not affect the cellular protein level of GCK compared to wild-type (Fig. 4C). When we further examined abundance using the DHFR-PCA, the mutants grew similar to wild-type GCK (Fig. 4D), again supporting that abundance was not affected. In conclusion, the region spanning residues 150–179 can be replaced by six residues (GSGSGS) or a 30-residue GS repeat without affecting GCK cellular abundance. This is consistent with the region being highly tolerant towards mutations in the super-open state.

If the super-open state is less destabilized than the closed state by mutations in the 150–179 region, variants are expected to shift the conformational equilibrium towards the super-open state, in turn resulting in decreased activity. Accordingly, re-stabilizing the closed state should increase activity. To test this, we focused on two residues in the 150–179 region, E157 and K161, that in the crystal structure of the closed state form an ion pair (Fig. 4E). Single mutants at these positions that reverse the charges, E157K and K161E, decrease activity but not abundance, based on their high-throughput assay scores (E157K activity = − 0.13, abundance = 0.96; K161E activity = 0.56, abundance= 0.92). A likely explanation is a conformational shift to an inactive state due to charge repulsion in the closed state. In turn, the closed state should become favorable again when reversing both charges using the double mutant E157K K161E, leading to increased activity relative to the single mutants. When we examined the activity of the mutants, the double mutant rescued the decreased activity of the single mutants (Fig. 4F), consistent with an increased population of the closed state.

Finally, inspired by previous work [30], we explored whether unbiased MD simulations could be used to gain further insight into how variants affect the conformational dynamics of GCK. We performed all-atom MD simulations of wild-type GCK and five GCK variants, four of which sit in the 150–179 region, starting from both closed and super-open conformations of the proteins (Additional file 1: Figs. S11 and S12). The simulations of wild-type GCK indicated that the small domain is more dynamic in the super-open conformation of the protein than in the closed conformation (Additional file 1: Figs. S13 and S14). However, we did not observe any substantial differences between the wild-type and variant dynamics for any of the simulated variants, at least not when evaluating variant effects on local fluctuations (Additional file 1: Figs. S15 and S16) and on deviations from wild-type crystal structures (Additional file 1: Fig. S14). Given the simulation time of 1 µs, we did not expect to observe an influence of variants on the slow structural transition between the closed and super-open states (Additional file 1: Fig. S17), since these transitions occur on a millisecond timescale [24]. However, our results show that even for very structurally destabilized variants and for variants with highly increased or decreased cellular activities, 1 µs of simulation is not enough to reveal how such variants might affect dynamics within the closed and super-open states, if those dynamics are actually affected.

Collectively, the above experiments support that variants in the 150–179 region decrease GCK activity by shifting the conformational ensemble towards inactive states. We cannot exclude that mutations in the region may cause local unfolding without affecting the global protein conformation. However, a prior study found that the 150–179 region folded in the absence of glucose when mutating the C-terminal helix 13 [24]. As other structural elements in the small domain affect the folding of the 150–179 region, it seems reasonable that variants causing the region to unfold would affect the entire domain’s conformation.

## Conclusions

Missense variants may perturb protein function through various mechanisms. Dissecting variant mechanisms allows us to gain insights into protein function and potentially to interfere with disease-causing variants. The development of multiplexed assays of variant effects (MAVEs) (also known as deep mutational scanning (DMS)) [45, 46] has enabled us to disentangle variant mechanisms on a massive scale by probing the effects of variants using multiple read-outs [7, 41, 47–51].

Building on our prior study on GCK variant activity [22], we here explored GCK variant mechanisms using a multiplexed assay reporting on cellular protein abundance. Our abundance scores included 95% of the possible nonsense and missense variants. Amino acid substitutions that decreased abundance were enriched in buried residues of the large domain. For this domain, loss of abundance therefore appears to be a general mechanism for loss-of-function variants. Accordingly, we find that 43% of variants that decrease activity do so together with abundance. The remaining 57% low-activity variants may instead perturb functional sites, such as catalytic residues, allosteric residues, or residues modulating GCK conformational dynamics.

Accordingly, in the dynamic small domain variants often perturbed activity but not abundance. This domain attains multiple conformations in GCK’s catalytic cycles [17], and these dynamics are crucial for appropriate GCK activity and regulation. Prior studies have focused mainly on hyperactive variants that affect the conformations and dynamics of GCK [22, 24–26]. For hypoactive variants, MD simulations have predicted five variants to shift GCK into the super-open inactive state [30]. Using predictions of changes in protein thermodynamic stability, we found that variants predicted to shift GCK into the inactive state are enriched in the 150–200 and 450 regions. In contrast to the prior molecular dynamics simulations and kinetic studies, however, using protein stability predictions allowed us to examine the conformational shift mechanism widely.

While computational predictions allowed us to broadly examine the conformational shift mechanism, we experimentally supported our findings focusing on the 150–179 region. We replaced the region by a small linker sequence without perturbing GCK’s cellular protein abundance, supporting that variants in the region are tolerated due to the region’s disorder in the super-open state. In turn, activity was rescued in a double mutant expected to stabilize the closed active state. Collectively, our results support that hypoactive variants may act by a relative destabilization of the closed state causing a conformational shift to the super-open inactive state.

In summary, we used a multiplexed abundance assay to identify variants that affect GCK protein stability and conformational dynamics. By identifying the mechanistic bases of hypoactive variants, we pinpointed the residues regulating stability and dynamics to ensure appropriate GCK activity. In turn, sites where such residues concentrate may be targeted to modulate GCK activity.

## Methods

### Buffers

SDS sample buffer (4×): 250 mM Tris/HCl, 40% glycerol, 8% SDS, 0.05% pyronin G, 0.05% bromophenol blue, pH 6.8. SDS sample buffer was diluted to 1.5× in water before use and 2% $\beta$-mercaptoethanol was added. TE buffer: 10 mM Tris/HCl, 1 mM EDTA, pH 8.0. PBS: 6.5 mM Na_2_HPO_4_, 1.5 mM KH_2_PO_4_, 137 mM NaCl, 2.7 mM KCl, pH 7.4. Wash buffer: 50 mM Tris/HCl, 150 mM NaCl, 0.01% Tween-20, pH 7.4.

### Plasmids

The DNA sequence of pancreatic human GCK (Ensembl ENST00000403799.8) was codon optimized for yeast and cloned into pDONR221 (Genscript). Selected missense variants were generated by Genscript. To generate a destination vector for the DHFR-PCA, a Gateway cassette was inserted 3′ to DHFR[F3] and a linker in pGJJ045 [34] (Genscript). *GCK* was cloned into the pDEST-DHFR-PCA destination vector using Gateway cloning (Invitrogen), such that the N-terminus of GCK was fused to DHFR[F3] (Additional file 1: Fig. S18). For the GCK activity assay, *GCK* was cloned into pAG416GPD-EGFP-ccdB (Addgene plasmid 14316; http://n2t.net/addgene:14316; RRID:Addgene_14316) [52] using Gateway cloning (Invitrogen).

### Yeast strains

BY4741 was used as the wild-type strain. The *hxk1*$\Delta$ *hxk2*$\Delta$ *glk1*$\Delta$ strain used for the GCK yeast complementation assay was generated previously [22]. Wild-type yeast cells were cultured in synthetic complete (SC) medium (2% D-glucose, 0.67% yeast nitrogen base without amino acids, 0.2% drop out (USBiological), (76 mg/L uracil, 76 mg/L methionine, 380 mg/L leucine, 76 mg/L histidine, 2% agar)) and Yeast extract-Peptone-Dextrose (YPD) medium (2% D-glucose 2% tryptone, 1% yeast extract). *hxk1*$\Delta$ *hxk2*$\Delta$ *glk1*$\Delta$ yeast cells were cultured in SC and YP medium containing D-galactose instead of D-glucose. Yeast transformations were performed as described before [53].

### Yeast growth assays

For growth assays, yeast cells were grown overnight and were harvested in the exponential phase (1200 g, 5 min, RT). Cell pellets were washed in sterile water (1200 g, 5 min, RT) and were resuspended in sterile water. The cultures were adjusted to an OD_600nm_ of 0.4 and were diluted using water in a fivefold serial dilution. The cultures were spotted in drops of 5 $\upmu$L onto agar plates. The plates were briefly air dried and were incubated at 30 ^∘^C (activity assay) or 37 ^∘^C (DHFR-PCA) for 2 to 4 days.

#### DHFR-PCA

To assay for GCK variant abundance, the DHFR-PCA was used [31–34]. For plates, SC medium with leucine, methionine, and histidine was used. For selection, a final concentration of 100 $\upmu$g/mL methotrexate (Sigma-Aldrich, 100 mM stock in DMSO) and 1 mM sulfanilamide (Sigma-Aldrich, 1 M stock in acetone) were used. For control plates, a corresponding volume of DMSO was used. Plates were incubated for 4 days at 37 ^∘^C. As a vector control for DHFR-PCAs, pAG416GPD-EGFP-ccdB was used.

#### GCK activity assay

To assay for GCK activity, yeast cells were grown on SC medium without uracil containing 0.2 % D-glucose for 3 days at 30 ^∘^C.

### Protein extraction

Yeast protein extraction was performed as described before [54]. Accordingly, 1.5–3 OD_600nm_ units of exponential yeast cells were harvested in Eppendorf tubes (17,000 g, 1 min, RT). Proteins were extracted by shaking cells with 100 $\upmu$L of 0.1 M NaOH (1400 rpm, 5 min, RT). Then, cells were spun down (17,000 g, 1 min, RT), the supernatant was removed, and pellets were dissolved in 100 $\upmu$L 1.5× SDS sample buffer (1400 rpm, 5 min, RT). Samples were boiled for 5 min prior to SDS-PAGE.

### Electrophoresis and blotting

To examine GCK protein levels, proteins in yeast extracts were separated by size on 12.5% acrylamide gels by SDS-PAGE. Subsequently, proteins were transferred to 0.2 $\upmu$m nitrocellulose membranes. Following western blotting, membranes were blocked in 5% fat-free milk powder, 5 mM NaN_3_, and 0.1% Tween-20 in PBS. Then, membranes were incubated overnight at 4 ^∘^C with a primary antibody diluted 1:1000. Membranes were washed 3 times 10 min with Wash buffer prior to and following a 1-h incubation with a peroxidase-conjugated secondary antibody. For detection, membranes were incubated for 2–3 min with ECL detection reagent (Amersham GE Healthcare) and were then developed using a ChemiDoc MP Imaging System (Bio-Rad). The primary antibody was anti-GFP (Chromotek, 3H9 3h9-100). The secondary antibody was HRP-anti-rat (Invitrogen, 31470).

#### Western blot quantification

To quantify protein levels from western blots, the Image Lab Software (Bio-Rad) was used. The software was used to measure the background-adjusted intensity of protein bands and the intensity of the Ponceau stain in the same lane. Then, a loading normalization factor was calculated for all lanes by dividing the ponceau intensity of lane 1 with that of all other lanes. Band intensities were adjusted by multiplying with their corresponding loading normalization factor. Finally, the loading-adjusted variant intensities were divided by the wild-type GCK intensity to obtain a normalized intensity that could be compared between replicates.

### Glucokinase library

#### Cloning

Three regional pENTR221 libraries spanning aa 2–171 (region 1), 172–337 (region 3), and 338–465 (region 3) of the GCK sequence were previously generated [22]. To clone the entry libraries into the DHFR-PCA destination vector, each regional entry library was used for a large-scale Gateway LR reaction consisting of: 169.6 ng pENTR221-GCK library, 450 ng pDEST-DHFR-PCA vector, 6 $\upmu$L Gateway LR Clonase II enzyme mix (ThermoFisher), TE buffer to 30 $\upmu$L. The LR reactions were incubated overnight (RT). The following day, each reaction was terminated by incubation with 3 $\upmu$L proteinase K (37 ^∘^C, 10 min). For each region, 4 $\upmu$L LR reaction was transformed into 100 $\upmu$L NEB 10-beta electrocompetent *E. coli* cells. Following electroporation, cells were recovered in NEB 10-beta outgrowth medium (37 ^∘^C, 1 h). Then, cells were plated on LB medium with ampicillin and incubated overnight at 37 ^∘^C. If at least 500,000 colonies were obtained, cells were scraped from plates using sterile water. Plasmid DNA was extracted from cells corresponding to 400 OD_600nm_ units (Nucleobond Xtra Midiprep Kit, Macherey-Nagel).

#### Yeast transformation

To express the GCK variant libraries in yeast, each regional plasmid library was transformed into the BY4741 yeast strain as described before [55] using the 30× scale-up. Briefly, yeast cells were grown overnight at 30 ^∘^C until late exponential phase. Cultures were then diluted with 30 ^∘^C YPD medium to an OD_600nm_ of 0.3 in a minimum volume of 150 mL and were incubated with shaking for 4–5 h until two divisions had occurred. Then, cells were harvested and washed two times in sterile water (1200 g, 5 min, RT). The cell pellet was resuspended in a transformation mix consisting of 7.2 mL 50% PEG, 1.08 mL 1.0 M LiAc, 300 $\upmu$L 10 mg/mL single-stranded carrier DNA, 30 $\upmu$g plasmid library, and sterile water to 10.8 mL. The cell suspension was incubated in a 42 ^∘^C water bath for 40 min with mixing by inversion every 5 min. Cells were harvested (3000 g, 5 min, RT), the supernatant was removed, and cells were resuspended in 30 mL sterile water. To assess the transformation efficiency, 5 $\upmu$L cells were plated in duplicate on SC-uracil medium. The remaining cells were diluted in SC-uracil medium to an OD_600nm_ of 0.2, and the cultures were incubated at 30 ^∘^C with shaking for 2 days until saturation.

If a minimum of 500,000 transformants were obtained, two cell pellets of 9 OD_600nm_ units were harvested (17,000 g, 1 min, RT) and stored at −20 ^∘^C prior to DNA extraction to serve as technical replicates of the pre-selection condition.

In parallel to the library transformations, pEXP-DHFR-PCA wild-type GCK was transformed into the BY4741 yeast strain using the small-scale transformation protocol [53].

#### Selection

To select for GCK variant abundance, the yeast libraries were grown in duplicate on medium containing 100 $\upmu$g/mL methotrexate and 1 mM sulfanilamide. For each regional yeast library, 20 OD_600nm_ units of cells were harvested in duplicate and were washed three times with sterile water (1200 g, 5 min, RT). The cells were resuspended in 500 $\upmu$L sterile water and each replicate was plated on a BioAssay dish (245 mm × 245 mm) containing SC+leucine+methionine+histidine medium with 100 $\upmu$g/mL methotrexate (Sigma-Aldrich) and 1 mM sulfanilamide (Sigma-Aldrich). The plates were incubated for 4 days at 37 ^∘^C. Following incubation, cells were scraped off each plate using 30 mL sterile water. Cell pellets of 9 OD_600nm_ units were harvested (17,000 g, 1 min, RT) and stored at − 20 ^∘^C prior to DNA extraction.

In parallel, yeast cells expressing pEXP-DHFR-PCA wild-type GCK were also used for selection as described above but using 2.6 OD_600nm_ units of yeast cells for each replicate, which were plated on petri dishes.

Plasmid DNA was extracted from yeast cells for two replicates pre- and post-selection, both for regional libraries and a wild-type GCK control. To extract plasmid DNA, the ChargeSwitch Plasmid Yeast Mini Kit (Invitrogen) was used.

#### Sequencing

In order to calculate the change in frequency of variants following selection, we sequenced the *GCK* ORF in plasmids extracted pre- and post-selection. Sequencing was done in 14 tiles spanning the *GCK *ORF, such that each regional library was covered by 4 or 5 tiles: region 1 (tiles 1–5), region 2 (tiles 6–10), and region 3 (tiles 10–14). The short tiles enabled sequencing of both strands in each tile to reduce base-calling errors. Although a limitation of using direct sequencing as a read-out is the risk of introducing PCR errors, the tileseqMave package (https://github.com/jweile/tileseqMave) used for data analysis accounts for the potential errors in multiple ways. First, amplicons derived from a wild-type GCK template were sequenced along with the non-selective and selective conditions, and variant frequencies observed in the wild-type control were subtracted from those observed in the non-selective and selective conditions. Second, the package performs quality filtering based on disagreement between the two replicates and variant counts in the non-selective condition.

First, the plasmid DNA extracted from yeast cells was adjusted to equal concentrations and was used for a PCR to amplify each tile. Each PCR consisted of 20 $\upmu$L Phusion High-Fidelity PCR Master Mix with HF Buffer (NEB), 1 $\upmu$L 10 $\upmu$M forward primer, 1 $\upmu$L 10 $\upmu$M reverse primer, 18 $\upmu$L plasmid library template. The following PCR program was used: 98 ^∘^C 30 s, 21 cycles of 98 ^∘^C 10 s, 63 ^∘^C 30 s, 72 ^∘^C 60 s, followed by 72 ^∘^C 7 min and 4 ^∘^C hold. Primer sequences can be found in the supplementary data (SKG_tilenumber_fw/rev).

Following tile amplification, Illumina index adapters were added to allow for multiplexing. For each indexing PCR, the following was mixed: 20 $\upmu$L Phusion High-Fidelity PCR Master Mix with HF Buffer (NEB), 2 $\upmu$L 10 $\upmu$M i5 indexing adapter, 2 $\upmu$L 10 $\upmu$M i7 indexing adapter, 1 $\upmu$L 1:10 diluted PCR product, 15 $\upmu$L nuclease-free water. The following PCR program was used: 98 ^∘^C 30 s, 7 cycles of 98 ^∘^C 15 s, 65 ^∘^C 30 s, 72 ^∘^C 120 s, followed by 72 ^∘^C 7 min and hold at 4 ^∘^C.

Following the indexing PCR, the indexed DNA fragments were pooled using equal volumes, and 100 $\upmu$L were run on a 4% E-gel EX Agarose Gel (Invitrogen) prior to gel extraction. Then, the quality and fragment size of the extracted DNA were examined using a 2100 Bioanalyzer system (Agilent), and the DNA concentration was adjusted using Qubit (ThermoFisher), before paired-end sequencing of the libraries using an Illumina NextSeq 550.

#### Data analysis

The TileSeqMave (https://github.com/jweile/tileseqMave, version 1.1.0) and TileSeq mutation count (https://github.com/RyogaLi/tileseq_mutcount, version 0.5.9) pipelines were used to calculate variant abundance scores from sequencing data.

#### Error calculation

Standard errors of abundance scores were calculated and refined using TileSeqMave (https://github.com/jweile/tileseqMave, version 1.1.0). In this pipeline, Bayesian refinement or regularization [56] is used to obtain more robust estimates of the standard errors. Briefly, linear regression of the fitness score and read counts from the pre-selection condition are used to obtain the prior estimate of the standard error. The empirical standard error is combined with the prior using Baldi and Long’s original formula, where $\sigma _0$ represents the prior estimate of the standard error, $v_0$ is the degrees of freedom given to the prior estimate, *n* represents the number of experimental replicates, and *s* is the empirical standard error:

$$\begin{aligned} \sigma ^2 = \frac{v_n\sigma _n^2}{v_n - 2} = \frac{v_0\sigma _0^2 + (n-1)s^2}{v_0 + n -2} \end{aligned}$$

### Computational analyses

#### Defining low-abundance threshold

To set a threshold for the abundance scores, we fitted the abundance score distribution using three Gaussians. These Gaussians represent the score distributions of variants with an abundance score comparable to nonsense variants, intermediate variants, and synonymous variants, respectively. To define a cutoff for variants with decreased abundance, we used the intersection of the second and last Gaussian.

#### Structure modeling and visualization

Protein structures were visualized and rendered using UCSF ChimeraX (v1.4), developed by the Resource for Biocomputing, Visualization, and Informatics at the University of California, San Francisco [57, 58]. The region spanning residues 157–179 missing from the crystal structure of the GCK super-open conformation (PDB: 1V4T) is shown in dashed lines in Fig. 4, but was modeled using Modeller [59] to be able to obtain $\Delta \Delta G$ estimates for variants in the region. Five structural models were generated with the Model Loops interface for Modeller available in ChimeraX (v1.3) using the super-open GCK structure (PDB: 1V4T) and the canonical GCK sequence (UniProt: P35557-1) as inputs. HETATM records and non-native terminal residues were removed from the PDB file using pdb-tools v2.4.3 [60] prior to the loop structure generation. Model Loops was run using the standard protocol, modeling only internally missing structure, and without allowing for any remodeling of residues adjacent to the missing segment.

#### Calculation of thermodynamic stability changes

Changes in protein thermodynamic stability ($\Delta \Delta G$ = $\Delta G_\text {variant} - \Delta G_\text {wildtype}$) caused by single-residue substitutions were predicted with Rosetta (GitHub sha1 c7009b3115 c22daa9efe2805d9d1ebba08-426a54) using the Cartesian ddG protocol [44]. Structure preparation and relaxation and the following $\Delta \Delta G$ calculations were performed using an in-house pipeline (https://github.com/KULL-Centre/PRISM/tree/main/software/rosetta_ddG_pipeline, v0.2.1). Rosetta $\Delta \Delta G$ output values were divided by 2.9 to convert from Rosetta energy units to kcal/mol [41, 44]. $\Delta \Delta G$ predictions for all possible point mutations in the segment spanning residues 157–179 were calculated for the super-open conformation of GCK based on the structural models created as described above. Predictions were performed for each of the five different structural models individually and subsequently averaged. For all analyses using $\Delta \Delta G$ predictions in this study, the values for the 157–179 region in the super-open conformation were used to supplement our previously reported $\Delta \Delta G$ values [22].

We performed additional $\Delta \Delta G$ calculations for this work using as input the structure of the closed GCK conformation bound to the glucose substrate, with glucose kept in the position observed in the crystal structure (PDB: 1V4S). $\Delta \Delta G$ value changes associated with the presence of the ligand were analyzed by comparing the results to our previous $\Delta \Delta G$ calculations, which were based on the closed conformation without substrate bound [22]. For the analysis, glucose binding site residues were defined as residues positioned less than 4 Å away from glucose, with the distance between each protein residue and glucose defined as the shortest distance between any heavy atom in glucose and the residue. Variants were defined as decreasing substrate binding affinity when the calculated variant $\Delta \Delta G$ was at least 2 kcal/mol larger in the glucose-bound structure than in the glucose-free structure.

#### Calculation of solvent accessible surface area and weighted contact number

The absolute solvent accessible surface area of every residue in both the closed (PDB: 1V4S) and super-open (PDB: 1V4T, with residues 157–179 added as described under the “Structure modeling and visualization” section) crystal structure conformations of GCK was calculated using DSSP [61] and normalized to a relative value (rASA) using a theoretically derived maximum accessibility per residue [62]. Residues with rASA $\le$ 0.2 were categorized as structurally buried, while residues with rASA > 0.2 were categorized as solvent-exposed. Moreover, a weighted contact number (WCN) was calculated for every residue *i* in the structures using the expression

1 $$\begin{aligned} \text {WCN}_{i} = \sum \limits _{j \ne i} s(r_{i,j}) \text {\quad with \quad } s(r)=\frac{1-\left( \frac{r}{r_0}\right) ^6}{1-\left( \frac{r}{r_0}\right) ^{12}}, \end{aligned}$$

where $r_{i,j}$ is the distance between residues *i* and *j*, and $r_0$ = 7 Å. Interresidue distances were evaluated using the MDTraj (v1.9.7, [63]) function compute_contacts. Distances were measured as the shortest distance between any interresidue pair of atoms for residue pairs involving glycine and as the shortest distance between any two sidechain heavy atoms for all other residue pairs.

### Molecular dynamics simulations

#### Preparation of starting structures

We performed atomistic molecular dynamics simulations of wild-type GCK and five GCK single substitution variants (D158A, G162Q, A173F, G175E, V455M) starting from both closed and super-open conformations of each variant. The simulation starting structures were prepared using the closed conformation crystal structure (PDB: 1V4S) and the super-open conformation crystal structure (PDB: 1V4T) with residues 157–179 added to the structure using Modeller [59], as described under the “Structure modeling and visualization” section. Mutations were introduced to the structures using the PyMOL (v2.5.2) Mutagenesis Wizard [64] by always selecting the mutant side chain rotamer with the smallest structural “strain.” In all structures, terminal charges were neutralized by capping with an acetyl group in the N-terminus and an amino group in the C-terminus, and non-native N-terminal residues present in the crystal structures were mutated to match the canonical GCK sequence (UniProt: P35557-1).

#### Simulation setup

All simulations were performed with Gromacs (v2021.1) [65] using the a99SB-disp force field in combination with the a99SBdisp water model [66]. Each system was prepared by placing the protein structure in the center of a dodecahedral box at least 1.1 nm from the box boundary. The protonation states of functional groups were set to match a pH value of 7.4 after evaluating the effective pKa values of all groups using ProPKA [67, 68]. The protonation site in singly protonated histidine residues was assigned automatically by Gromacs. After solvation with water molecules, counter ions were added to neutralize the system and additional ions (Na^+^ and Cl^-^) were added to reach an ion concentration of 0.15 M. Na^+^ ions originally present in the crystal structures were kept.

System preparation was followed by energy minimization by steepest descent with an initial minimization step size of 0.01 nm and until the maximum system force was smaller than 1000 kJ mol^-1^ nm^-1^. A four-step equilibration protocol was then applied to make sure that all protein structures were stable in the simulations. First, the thermostat target temperature was linearly increased from 10 to 200 K during a 5-ns NVT simulation in which position restraints with a force constant of 1000 kJ mol^-1^ nm^-2^ were applied to all protein heavy atoms. Second, the target temperature was further linearly increased from 200 to 310 K during another 5-ns NVT simulation with position restraints only on the protein C$\alpha$ atoms. Third, all position restraints were dropped, and pressure was equilibrated to 1 bar using the Berendsen barostat [69] in a 3-ns NPT simulation. Fourth, equilibration was finalized by switching to the Parrinello-Rahman barostat [70] and extending the simulation time with 3 ns. Following equilibration, production simulations were run for 1 µs at 310 K and 1 bar, saving the system conformation every 100 ps. All simulations were run in replicas of three (different random velocities were used to initialize each replica) producing three trajectories for every GCK variant in each GCK starting conformation.

In all simulations, covalent bonds involving hydrogen atoms were constrained with the LINCS algorithm [71]. Van der Waals interactions and short-range electrostatic interactions were evaluated with a 1.2-nm cutoff [66]. For van der Waals interactions, the force was shifted and smoothly switched to zero between 1 and 1.2 nm. The neighbor list was updated at least every 10th step. Long-range electrostatic interactions were calculated with the smooth particle mesh Ewald method [72] with a grid spacing of 0.16 nm and an interpolation order of four. The leap-frog algorithm was used for integration with a timestep of 2 fs. For both equilibration and production, temperature was controlled with the velocity-rescaling thermostat [73], treating protein and non-protein atoms as separate coupling groups, and with a coupling time constant of 1 ps. Pressure coupling was applied with a time constant of 2 ps and setting compressibility to 4.5$\cdot 10^{-5}$ bar^-1^ for both the Berendsen and Parrinello-Rahman barostats.

#### Trajectory analysis

Production trajectories were analyzed with Gromacs (v2021.1) [65] and MDTraj (v1.9.7) [63]. Raw trajectory files were processed with the Gromacs tool *trjconv* to center the protein in the simulation box and to remove non-protein species prior to further analysis. MDTraj was used to calculate the backbone heavy atom RMSD between all simulated structures and the wild-type GCK crystal structures. The RMSD calculations were carried out separately for each protein domain, with domain definitions used for the simulation analysis specified in Additional file 2. RMSF values were similarly evaluated for each domain with MDTraj from the fluctuations of C${\alpha }$ atoms around the average simulation structures. Angles and distances between atoms were also calculated with MDTraj tools. The cleft angle between the small and large domains of GCK was monitored by calculating the angle between the C${\alpha }$ atoms of residues 109, 233, and 229, while the movement of helix 13 was studied by calculating the distance between the C${\alpha }$ atoms of residues 159 and 452. The cleft angle definition was inspired by previous work in which the 159–452 distance was also introduced and monitored [30].

### Supplementary information

**Additional file 1.** Supporting information for: Characterizing glucokinase variant mechanisms using a multiplexed abundance assay, supplementary figures.

**Additional file 2.** Supplementary data, all data presented in the manuscript.

**Additional file 3.** Review history.

## Data Availability

The datasets supporting the conclusions of this article are available in Additional file 2. In addition, sequencing reads are available at the NCBI Gene Expression Omnibus (GEO) repository (accession number GSE226732, https://www.ncbi.nlm.nih.gov/geo/query/acc.cgi?acc=GSE226732) [74] and abundance scores have been deposited in the MaveDB repository (accession number urn:mavedb:00000096-b, https://www.mavedb.org/#/experiments/urn:mavedb:00000096-b) [75]. The activity scores that were generated previously are available at Zenodo (https://doi.org/10.5281/zenodo.7636310) [76]. The code for generating the plots found in the paper is available at GitHub (https://github.com/KULL-Centre/_2024_Gersing_GCKabundance) with an MIT license [77]. The GitHub repository is also available at Zenodo (https://doi.org/10.5281/zenodo.10837281) [78]. The GitHub page also contains scripts for analysis of simulation data as well as input files and example scripts for starting simulations. Processed simulation data is available at https://doi.org/10.17894/ucph.4e38c597-fae2-4654-a48a-8e714263c5a1.
