# Supplementary material for: Characterizing glucokinase variant mechanisms using a multiplexed abundance assay
Source: Genome Biol. 2024 Apr 16;25:98. doi: 10.1186/s13059-024-03238-2 (PMC11021015; doi:10.1186/s13059-024-03238-2)
Supplement: Supplementary file 1 — Additional file 1. Supporting information for: Characterizing glucokinase variant mechanisms using a multiplexed abundance assay, supplementary figures. [file 13059_2024_3238_MOESM1_ESM.pdf]

Supporting information for: Characterizing glucokinase variant mechanisms using a multiplexed abundance assay

Sarah Gersing[1*], Thea K. Schulze[1], Matteo Cagiada[1], Amelie Stein[1], Frederick P. Roth[2,3,4,5], Kresten Lindorff-Larsen[1*], Rasmus Hartmann-Petersen[1*]

***For correspondence:**
sarah.gersing@bio.ku.dk (S.G.);
lindorff@bio.ku.dk (K.L.-L.);
rhpetersen@bio.ku.dk (R.H.-P.)

[1]The Linderstrøm-Lang Centre for Protein Science, Department of Biology, University of Copenhagen, Ole Maaløes Vej 5, DK-2200 Copenhagen, Denmark; [2]Donnelly Centre, University of Toronto, Toronto, ON, M5S 3E1, Canada; [3]Department of Molecular Genetics, University of Toronto, Toronto, ON, M5S 1A8, Canada; [4]Lunenfeld-Tanenbaum Research Institute, Sinai Health, Toronto, ON, M5G 1X5, Canada; [5]Department of Computational and Systems Biology, University of Pittsburgh School of Medicine, Pittsburgh, USA 15213

Contents

S1 Abundance scores of test variants and correlation with western blot protein levels. . 3
S2 Correlation of abundance scores with weighted contact numbers. 3
S3 Defining a threshold for low-abundance variants. 4
S4 Score distributions for the two domains. 5
S5 Correlations of variant activity scores with GEMME and $\Delta\Delta G$ scores. 6
S6 Correlations of residue median activity scores with GEMME and $\Delta\Delta G$ scores. 7
S7 Correlations of variant abundance scores with GEMME and $\Delta\Delta G$ scores. 8
S8 Correlations of residue median abundance scores with GEMME and $\Delta\Delta G$ scores. . . 9
S9 The activity and abundance scores of GCK-MODY variants. 10
S10 Identifying residues and variants where glucose affects $\Delta\Delta G$ predictions. 10
S11 List of variants selected for being studied with MD simulations. 11
S12 Structural visualisation of variants simulated with MD. 11
S13 Differences in dynamics between closed and super-open GCK conformations. 12
S14 Average RMSD between simulated GCK conformations and crystal structures. 13
S15 Dynamics in the closed conformation. 14
S16 Dynamics in the super-open conformation. 15
S17 Average values of collective variables reporting on transition between the closed and super-open conformations. 16

S18 Plasmid map of pEXP-DHFR-PCA-GCK. 17
Supplemental references 18

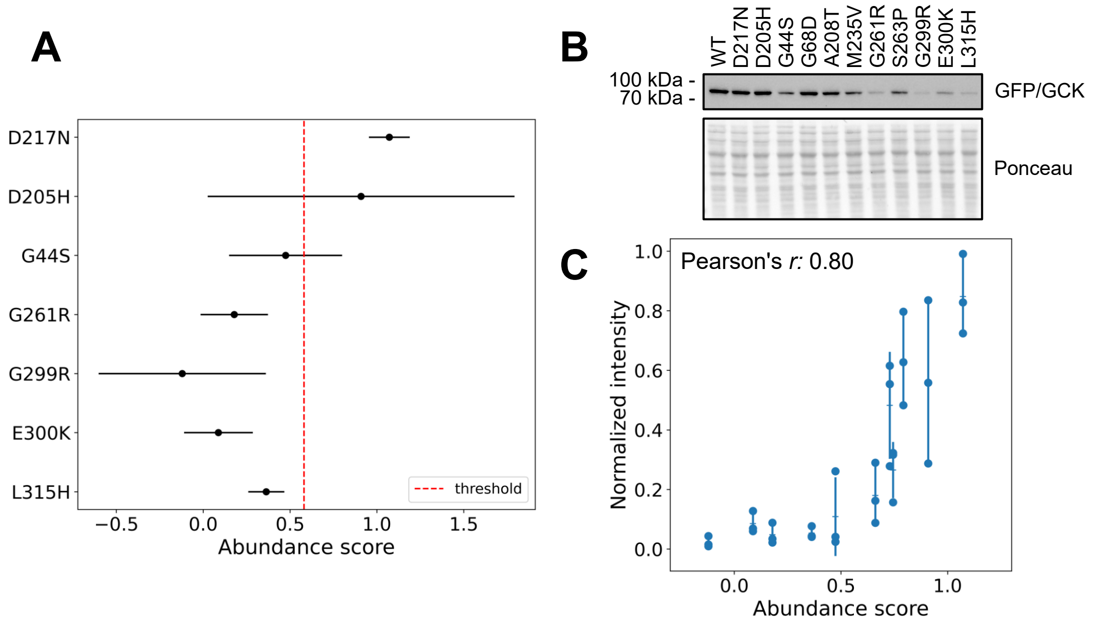

Figure S1. Abundance scores of test variants and correlation with western blot protein levels. (A) The abundance score and standard error of the initial test variants. The threshold for low abundance is below a score of 0.58. (B) Western blot showing the protein levels of selected GCK variants spanning a wide range of abundance scores. (C) The protein levels (normalized intensity) of the 11 variants shown in panel B were quantified from three western blots. Points indicate individual western blot quantifications, horizontal lines show the mean protein level of each variant, and vertical lines show the standard deviation. The Pearson correlation between the abundance scores and three western blot quantifications is 0.80.

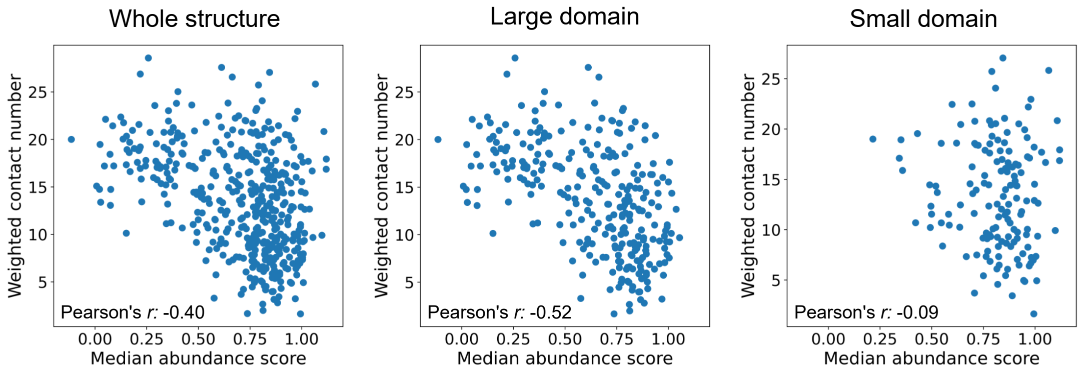

Figure S2. Correlation of abundance scores with weighted contact numbers. The plots show the correlation between the weighted contact number and the median abundance score for each residue. The weighted contact numbers were calculated using the closed state of GCK (PDB: 1V4S), and are a measure of residue burial, such that a higher weighted contact number indicates that a given residue is more buried. The correlation is shown for all residues in the structure and for residues in the large and small domain. For the large domain, a lower median abundance is associated with a higher contact number, suggesting that mutations at buried residues decrease abundance.

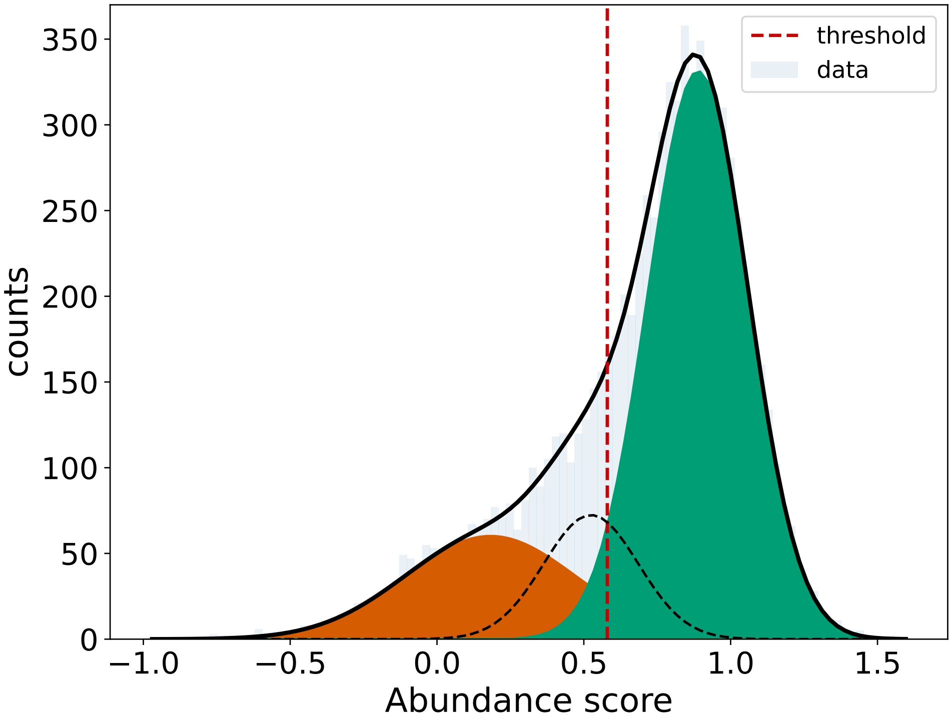

Figure S3. Defining a threshold for low-abundance variants. We defined a threshold for low-abundance variants by fitting the abundance score distribution (light blue) using three Gaussian distributions. We used the intersection between the second (dashed line) and last Gaussian (green) to define the cutoff (0.58, red dashed line) for variants with decreased abundance.

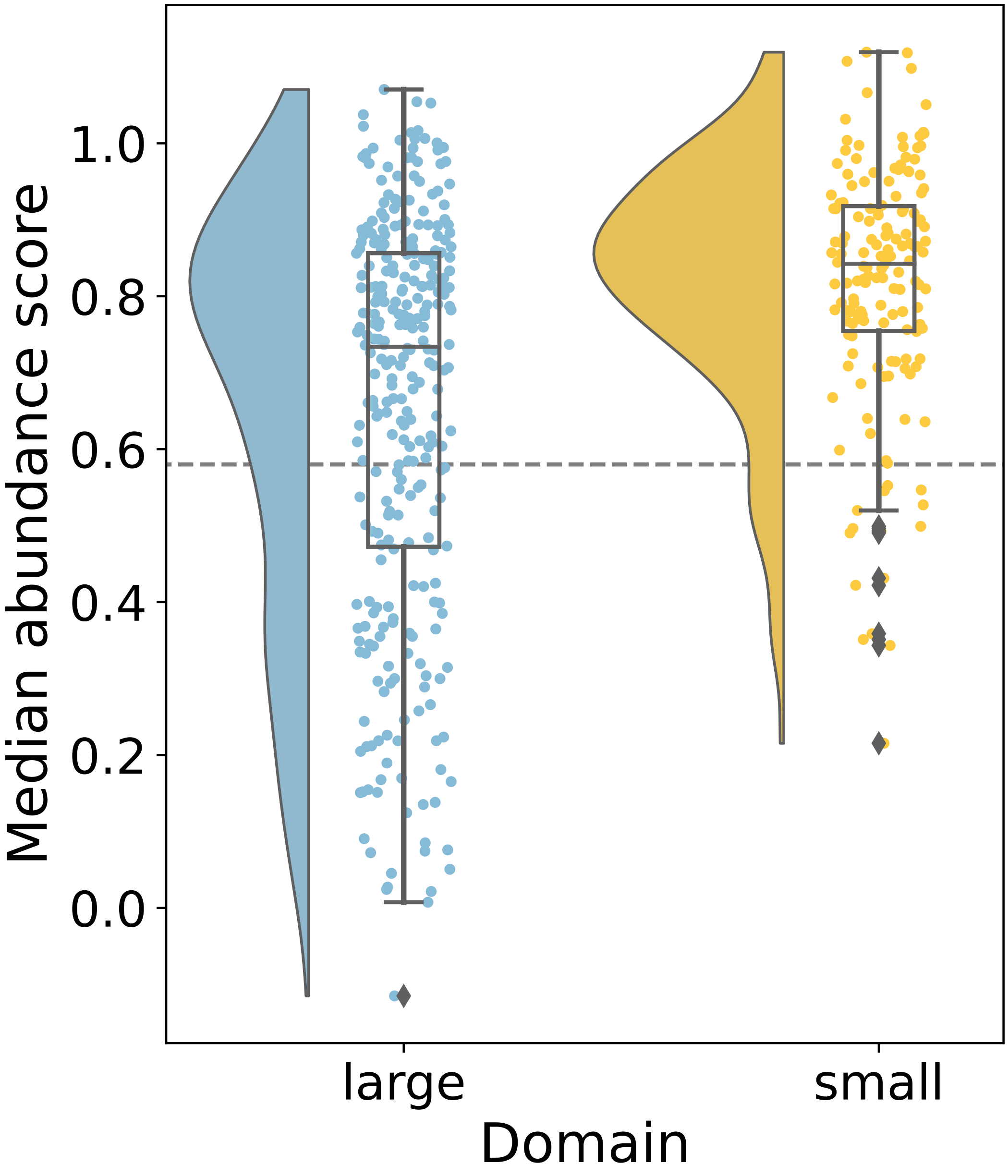

Figure S4. Score distributions for the two domains. The distributions of median abundance scores for residues belonging to the large or the small domain. The dashed line indicates the threshold for decreased abundance (0.58).

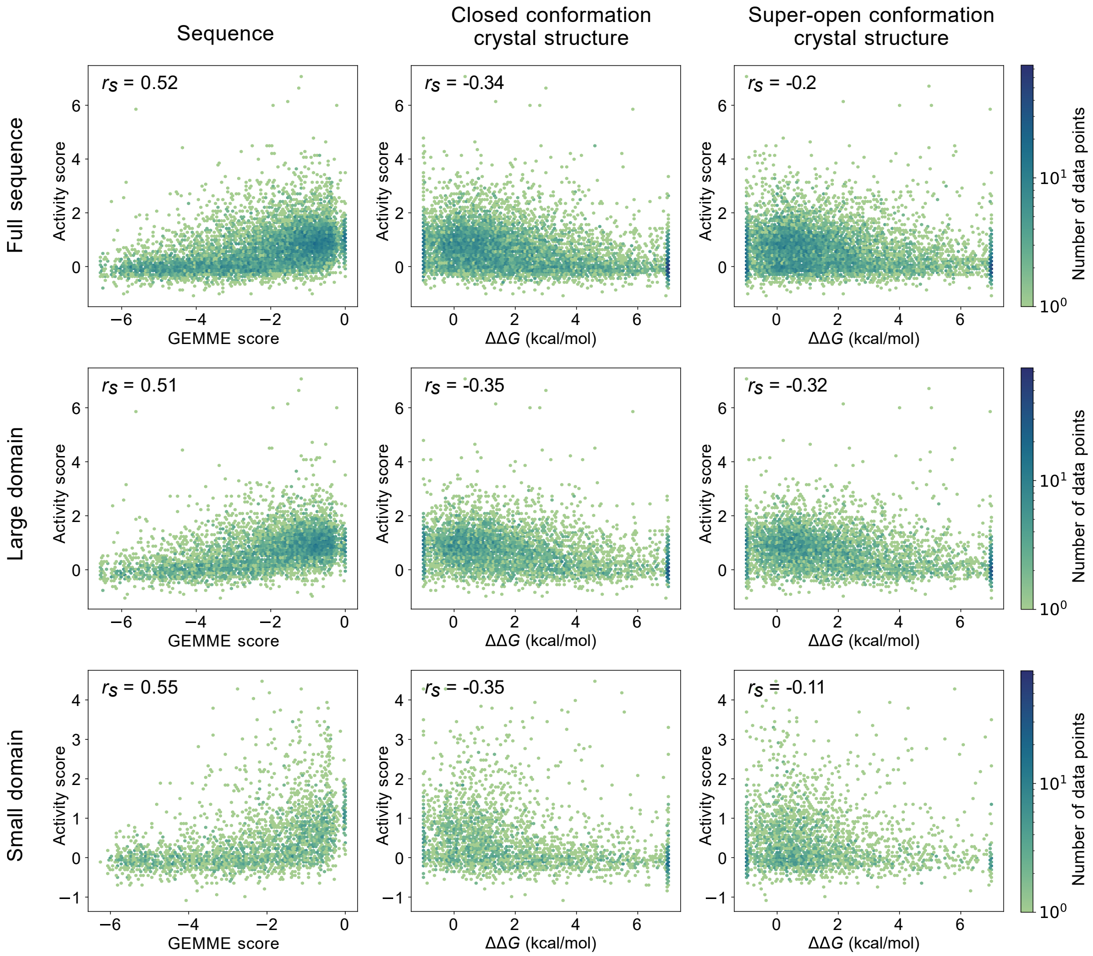

Figure S5. Correlations of variant activity scores with GEMME and $\Delta\Delta G$ scores. Plots showing the correlations of variant activity scores with evolutionary distance scores (GEMME score) and predicted changes in protein thermodynamic stability ($\Delta\Delta G$) for the full glucokinase sequence, the large domain and the small domain. Spearman's correlation coefficients (r_s) are shown in each plot. Activity scores, GEMME scores and $\Delta\Delta G$ values were obtained from [1].

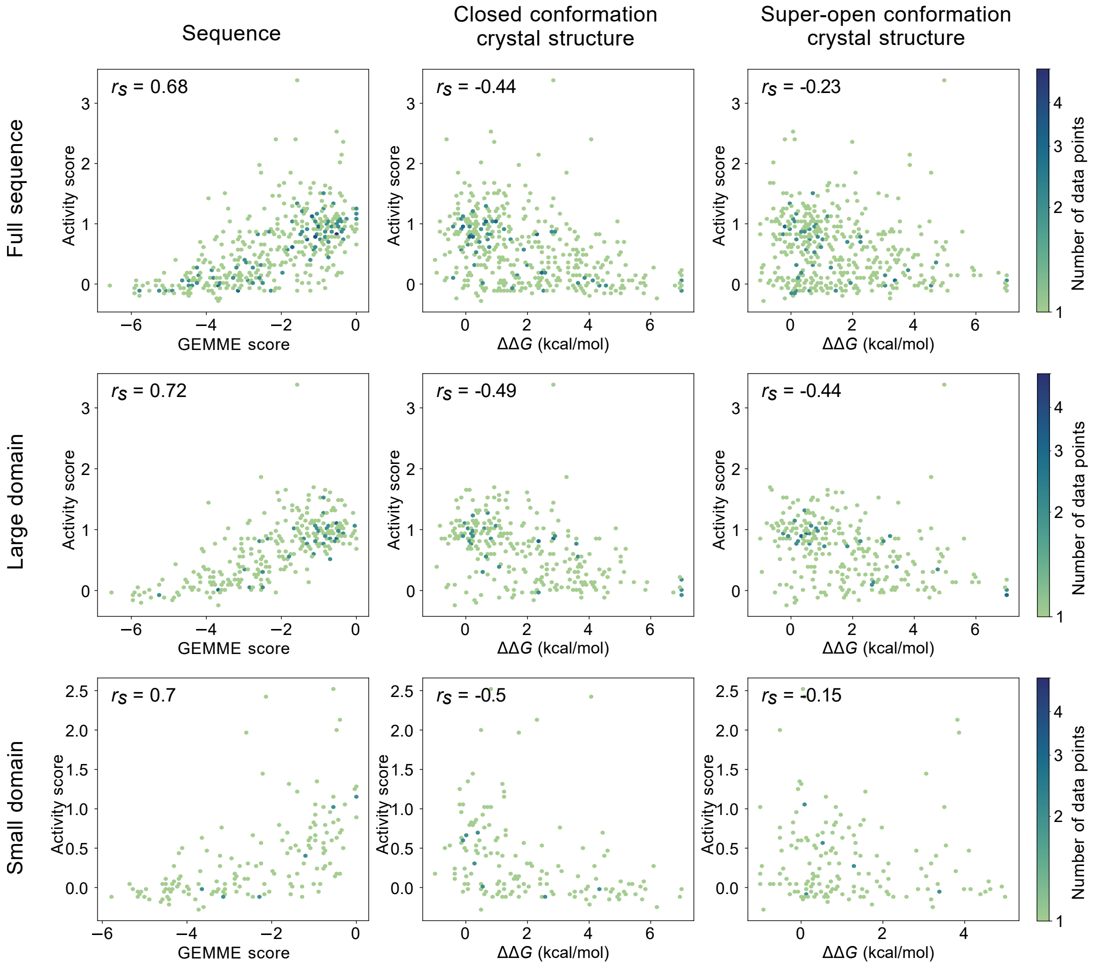

Figure S6. Correlations of residue median activity scores with GEMME and $\Delta\Delta G$ scores. Plots showing the correlations of residue median activity scores with evolutionary distance scores (GEMME score) and predicted changes in protein thermodynamic stability ($\Delta\Delta G$) for the full glucokinase sequence, the large domain and the small domain. Spearman's correlation coefficients (r_s) are shown in each plot. Activity scores, GEMME scores and $\Delta\Delta G$ values were obtained from [1].

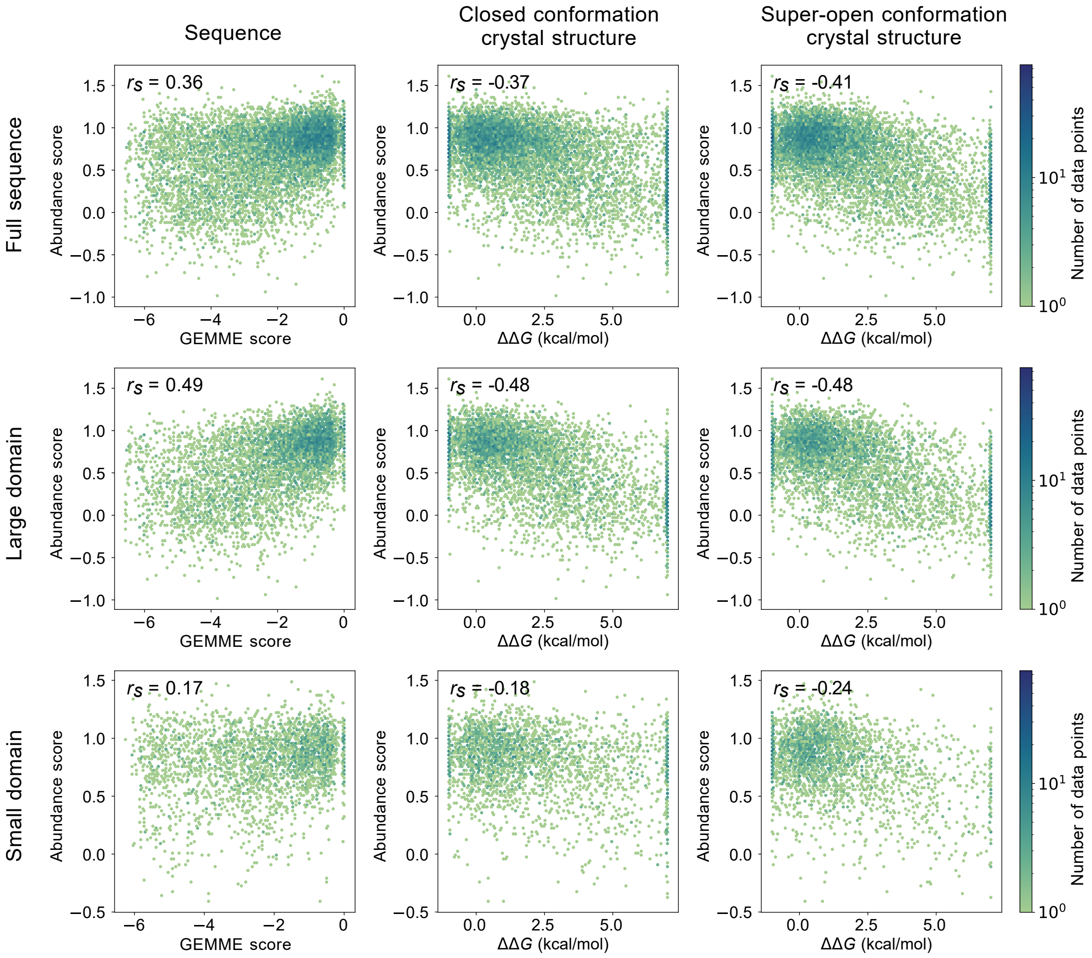

Figure S7. Correlations of variant abundance scores with GEMME and $\Delta\Delta G$ scores. Plots showing the correlations of variant abundance scores with evolutionary distance scores (GEMME score) and predicted changes in protein thermodynamic stability ($\Delta\Delta G$) for the full glucokinase sequence, the large domain and the small domain. Spearman's correlation coefficients (r_s) are shown in each plot. GEMME scores and $\Delta\Delta G$ values were obtained from [1].

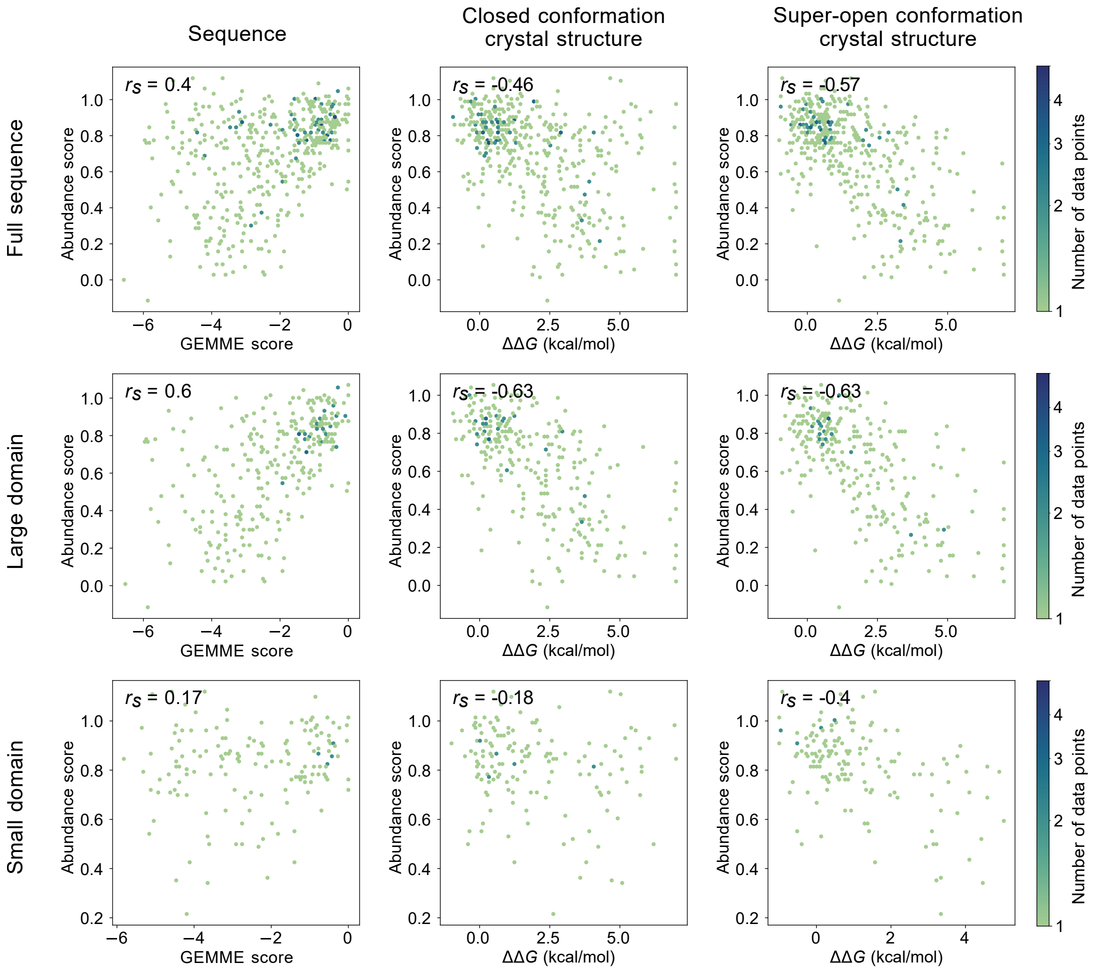

Figure S8. Correlations of residue median abundance scores with GEMME and $\Delta\Delta G$ scores. Plots showing the correlations of residue median abundance scores with evolutionary distance scores (GEMME score) and predicted changes in protein thermodynamic stability ($\Delta\Delta G$) for the full glucokinase sequence, the large domain and the small domain. Spearman's correlation coefficients (r_s) are shown in each plot. GEMME scores and $\Delta\Delta G$ values were obtained from [1].

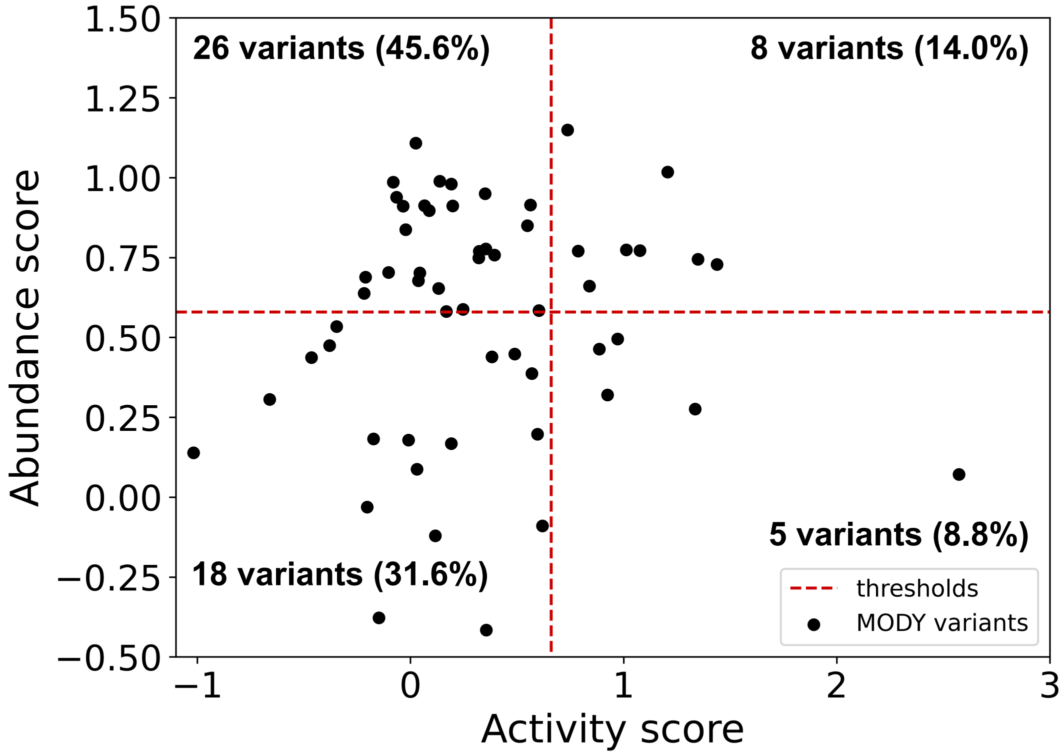

Figure S9. The activity and abundance scores of GCK-MODY variants. Plot showing the activity and abundance scores of 57 variants associated with GCK-MODY. The list of GCK-MODY variants were obtained from [2], and only variants with both scores were included. The thresholds for low activity (0.66) and low abundance (0.58) are shown as red dashed lines.

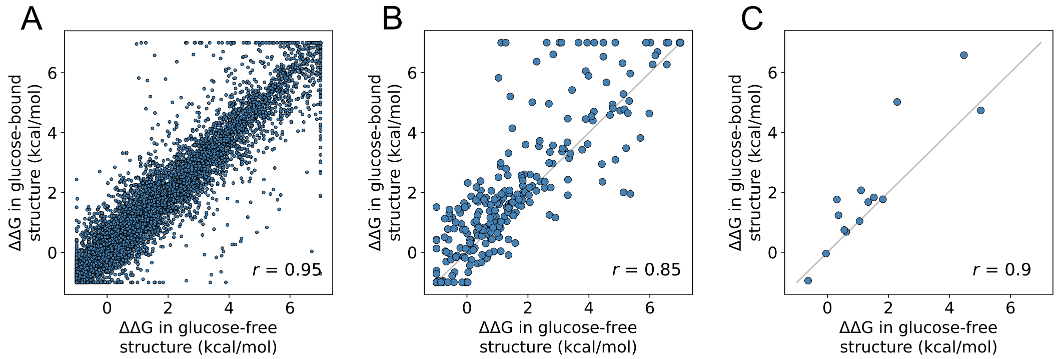

Figure S10. Identifying residues and variants where glucose affects $\Delta\Delta G$ predictions. (A) Plot showing the correlations of $\Delta\Delta G$ values for all variants predicted using the closed GCK structure with and without glucose bound. (B) Plot showing the same as in A, but only including variants of residues in the glucose binding site. Residues 229, 230, 231 and 256 have at least a single variant where the $\Delta\Delta G$ is more than 2 kcal/mol larger in the glucose-bound structure than in the glucose-free structure. Individual variants predicted to decrease ligand binding affinity are listed in additional file 2. (C) The correlation of median $\Delta\Delta G$ values per residue predicted using the glucose-bound and glucose-free structure of GCK and only including residues in the glucose binding site. Residues 229 and 231 have median $\Delta\Delta G$ values that are more than 2 kcal/mol larger in the glucose-bound structure than in the glucose-free structure. In all plots the Pearson correlation coefficient (r) is shown.

Variant	Activity score	Abundance score	ΔΔΔG (kcal/mol)	$\Delta\Delta G_{superopen}$ (kcal/mol)	$\Delta\Delta G_{closed}$ (kcal/mol)
D158A	1.71	1.01	-0.61	0.70	1.31
G162Q	-0.16	0.94	-5.87	0.11	5.98
A173F	-0.23	0.94	-11.29	-0.26	11.0
G175E	-0.065	0.94	-0.95	0.61	1.57
V455M	2.48	1.42	2.03	3.04	1.01

Figure S11. List of variants selected for being studied with MD simulations. Experimental activity [1] and abundance scores as well as calculated $\Delta\Delta G$ scores for the five variants for which molecular dynamics simulations were performed. The list of variants include both hypo- and hyperactivating variants as well as variants predicted to have different impacts on the ΔG. The G175E and V455M variants are associated with MODY and HH, respectively [2].

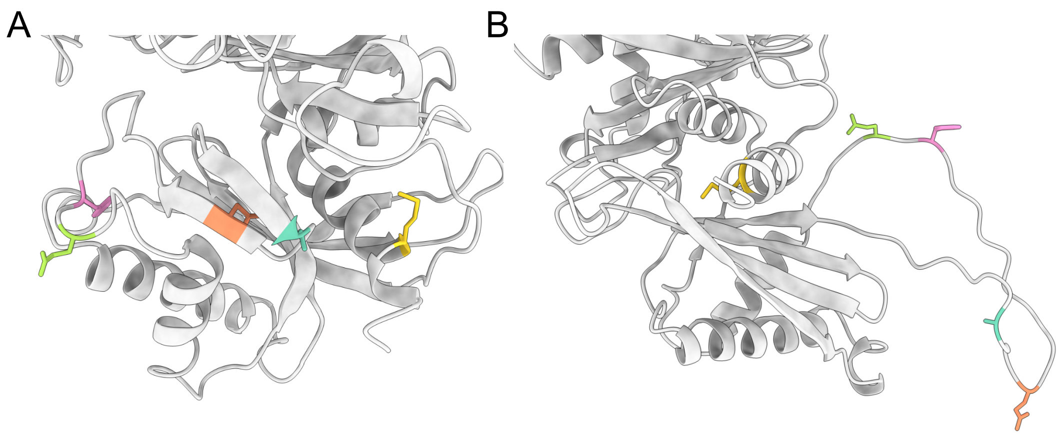

Figure S12. Structural visualisation of variants simulated with MD. (A) Small domain in the closed conformation with all variant residue side chains highlighted simultaneously. (B) Small domain in the super-open conformation, similarly with all variant residue side chains highlighted at the same time. The super-open structure in B is rotated 180 degrees with respect to the closed structure in A. Residues are coloured according to the colour scheme used for variants in Fig. S15 and S16, with D158A in cyan, G162Q in orange, A173F in pink, G175E in green and V455M in yellow. The side chain conformations shown here correspond to the initial side chain conformations in the MD simulations. Visualisations were created with ChimeraX [3, 4].

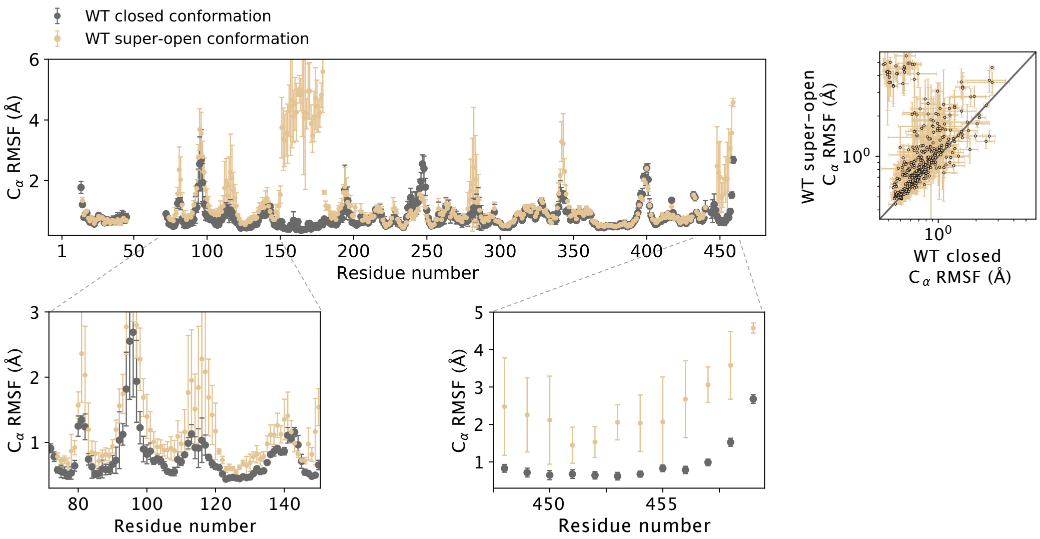

Figure S13. Differences in dynamics between closed and super-open GCK conformations. Cα root-mean-square fluctuation (RMSF) values calculated from MD simulations of wild-type GCK starting from either the closed or super-open conformation of the protein. RMSFs were calculated separately for each protein domain and with respect to the average domain structure in each simulation. Some data points are missing because the corresponding residues were not assigned to be in any folded domain, but instead to be in hinge regions. To the left, RMSF values are shown as a function of residue number, with bottom plots highlighting RMSFs of residues sitting in the small domain. Reported RMSFs are averages across three simulation replicas, and error bars are standard deviations over the three individual simulation averages. To the right, values from closed and super-open conformations are plotted against each other.

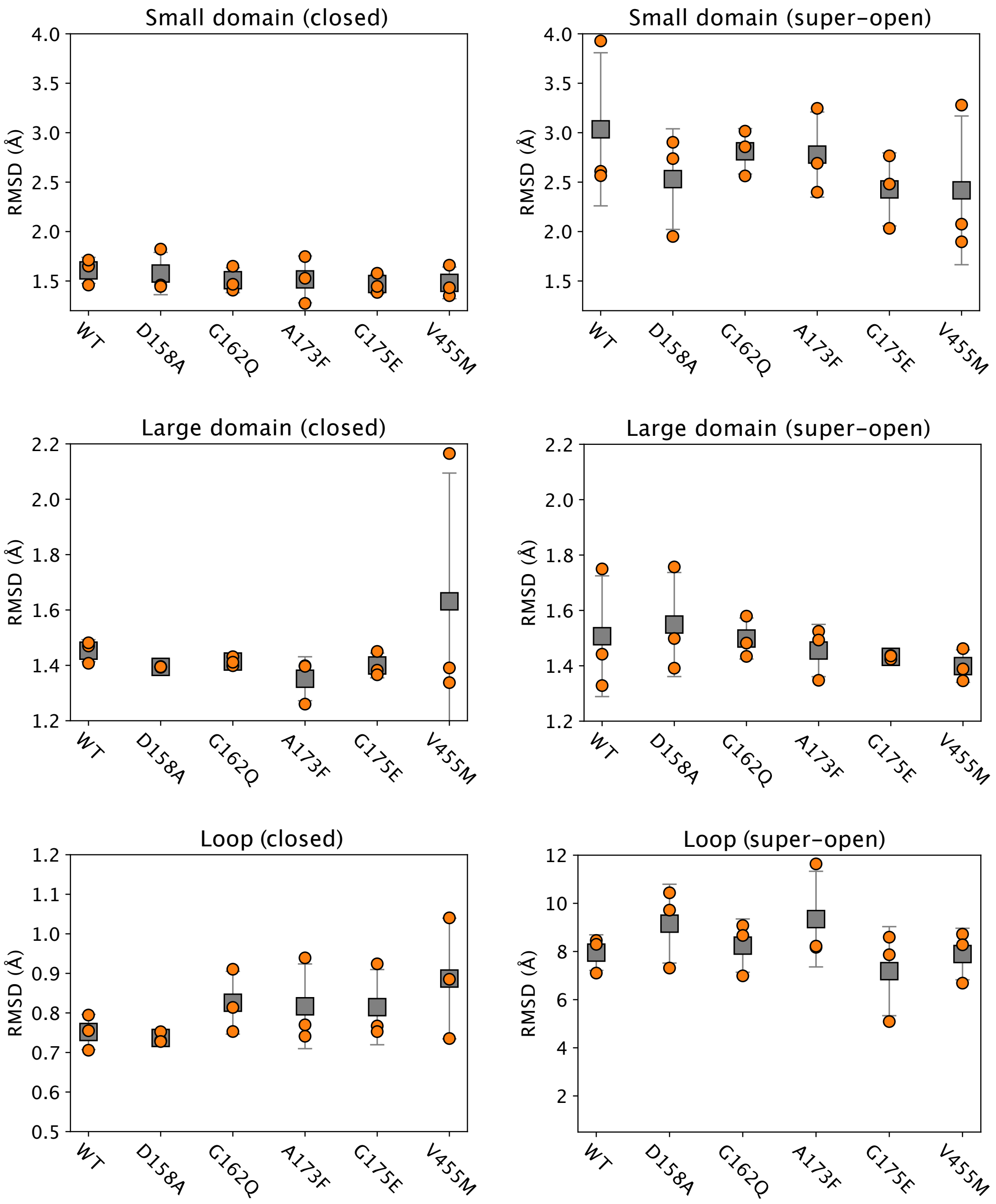

Figure S14. Average RMSD between simulated GCK conformations and crystal structures. Root-mean-square deviation (RMSD) values for GCK wild-type and the five variants were calculated from simulations started from the closed (left) and super-open (right) crystal structure conformations using the relevant wild-type crystal structure as reference, also for evaluation of variants. RMSD calculations were done separately for each domain, with loop referring to residues 151–179. Orange circles are average values from single simulation replicas. Gray squares are averages across three replicas, and error bars are standard deviations across the three replica averages. Note the changing y-axis values.

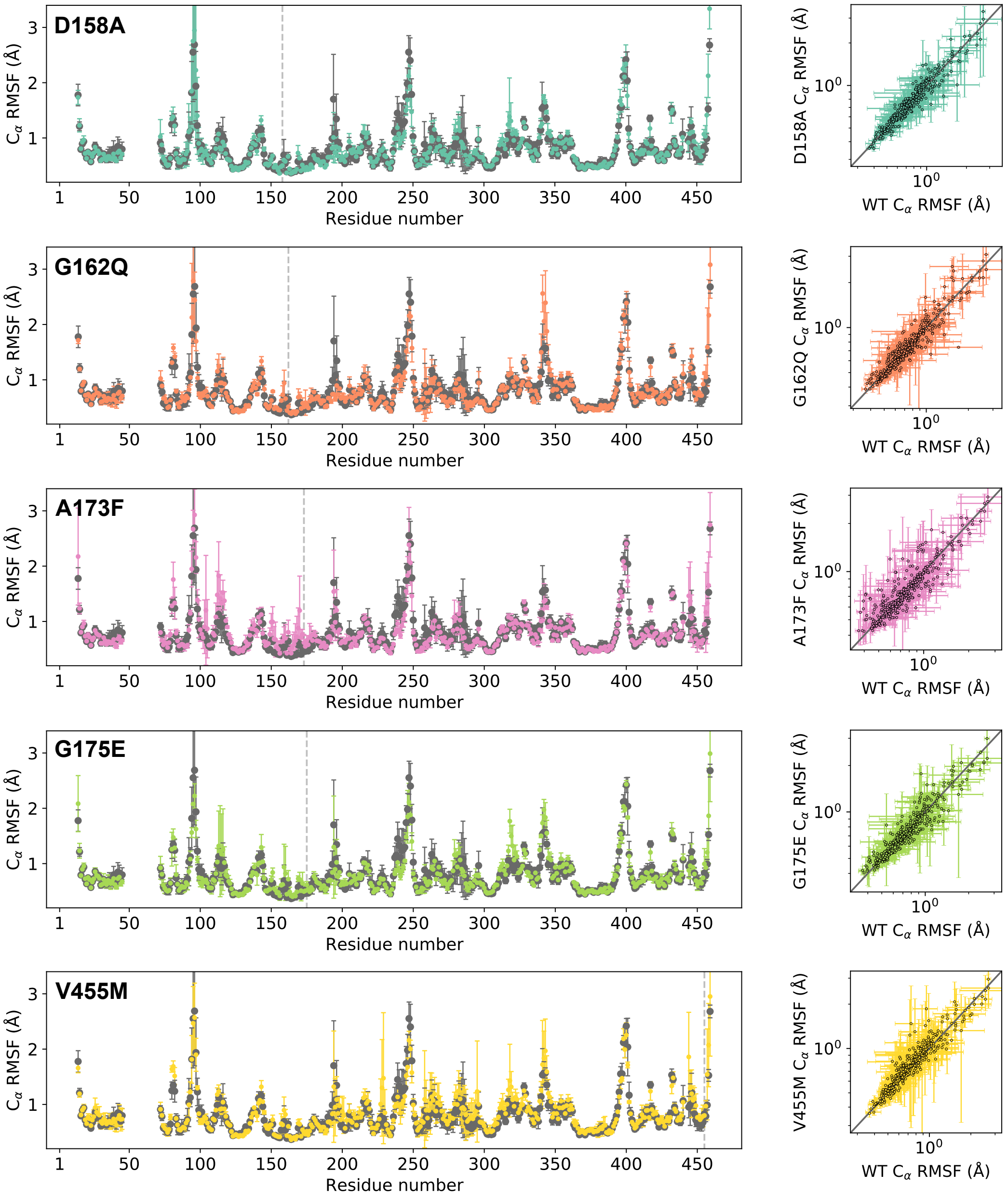

Figure S15. Dynamics in the closed conformation. $C\alpha$ root-mean-square fluctuation (RMSF) values calculated from wild-type and variant MD simulations in which starting structures were based on the closed conformation of GCK. RMSFs were calculated separately for each protein domain and with respect to the average domain structure in each simulation. Some data points are missing because the corresponding residues were not assigned to be in any folded domain, but instead to be in hinge regions. To the left, RMSF values are shown as a function of residue number. Gray data points are RMSFs calculated from wild-type GCK simulations, and coloured data points are RMSFs calculated from variant trajectories. Reported RMSFs are averages across three simulation replicas, and error bars are standard deviations over the three individual simulation averages. Dashed vertical lines indicate the position of the variant in each plot. To the right, the wild-type and the variant values are plotted against each other to underline the high correlations between the data points.

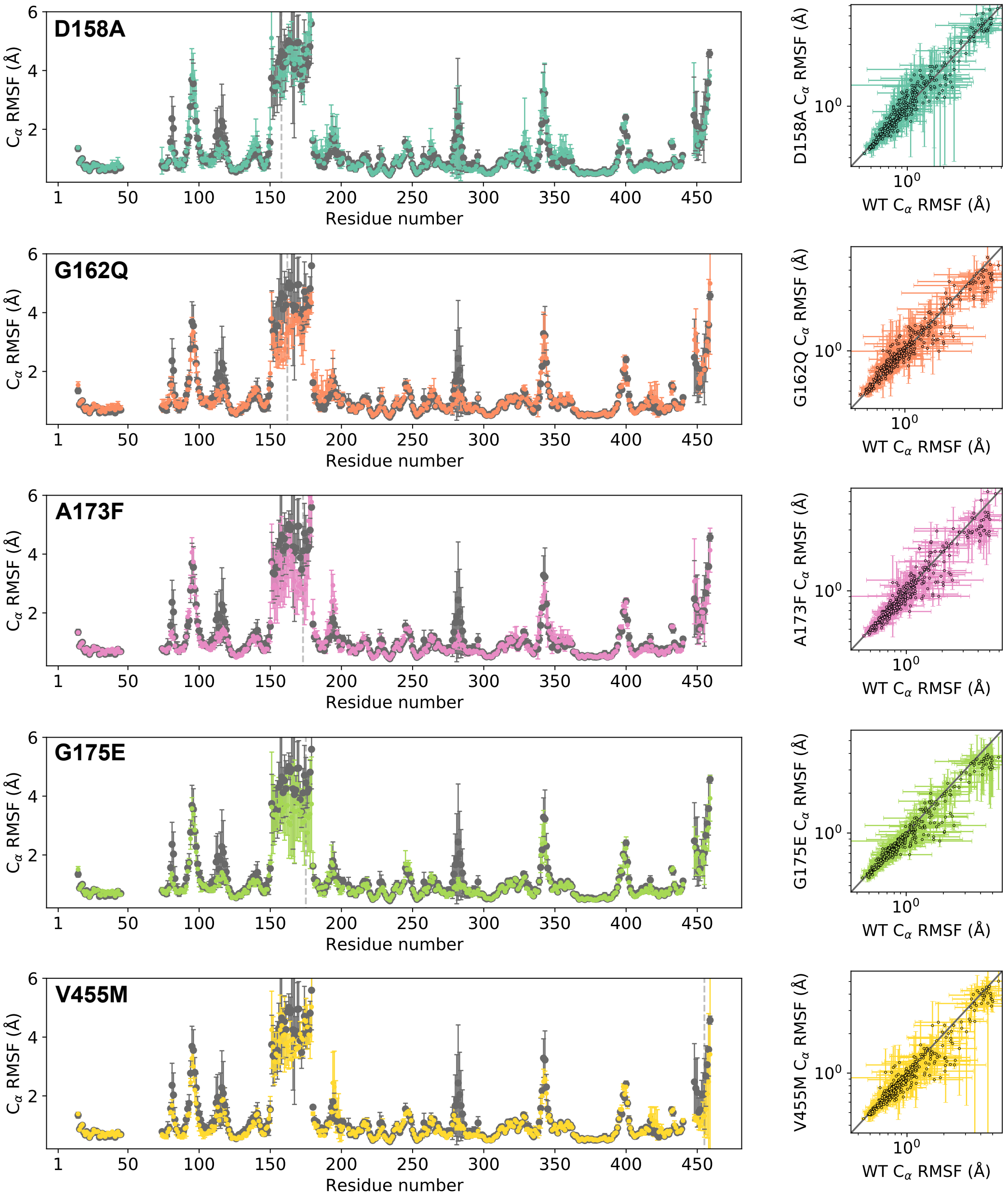

Figure S16. Dynamics in the super-open conformation. Cα root-mean-square fluctuation (RMSF) values calculated from wild-type and variant MD simulations in which starting structures were based on the super-open conformation of GCK. RMSFs were calculated separately for each protein domain and with respect to the average domain structure in each simulation. Some data points are missing because the corresponding residues were not assigned to be in any folded domain, but instead to be in hinge regions. To the left, RMSF values are shown as a function of residue number. Gray data points are RMSFs calculated from wild-type GCK simulations, and coloured data points are RMSFs calculated from variant trajectories. Reported RMSFs are averages across three simulation replicas, and error bars are standard deviations over the three individual simulation averages. Dashed vertical lines indicate the position of the variant in each plot. To the right, the wild-type and the variant values are plotted against each other to underline the high correlations between the data points.

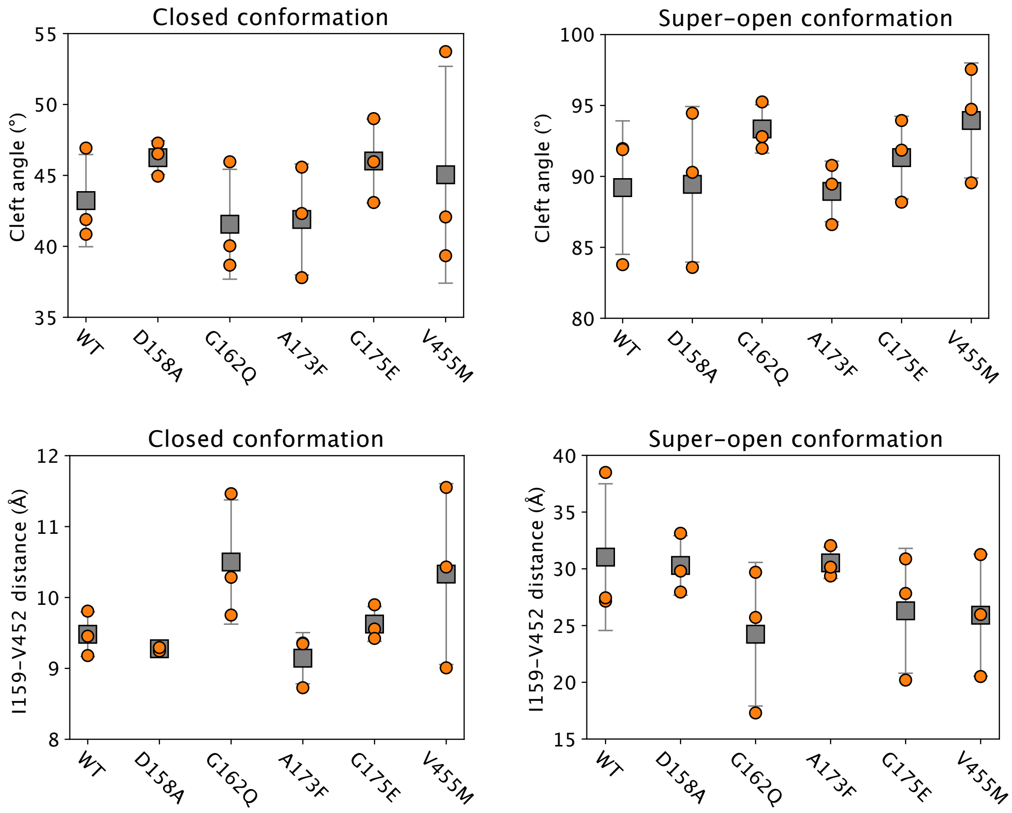

Figure S17. Average values of collective variables reporting on transition between the closed and super-open conformations. (*Top*) The cleft angle, describing the size of the angle between the large and small domain, was calculated for all GCK wild-type and variant simulations in both closed and super-open conformations. (*Bottom*) The distance between residues I159 and V452, reporting on the position of helix 13, was similarly calculated for all simulations. Orange circles are average values from single simulation replicas. Gray squares are averages across three replicas, and error bars are standard deviations across the three replica averages. Note the changing y-axis values. The definition of the cleft angle was inspired by previous work which also introduced the I159-V452 distance to monitor GCK opening from the closed state [5].

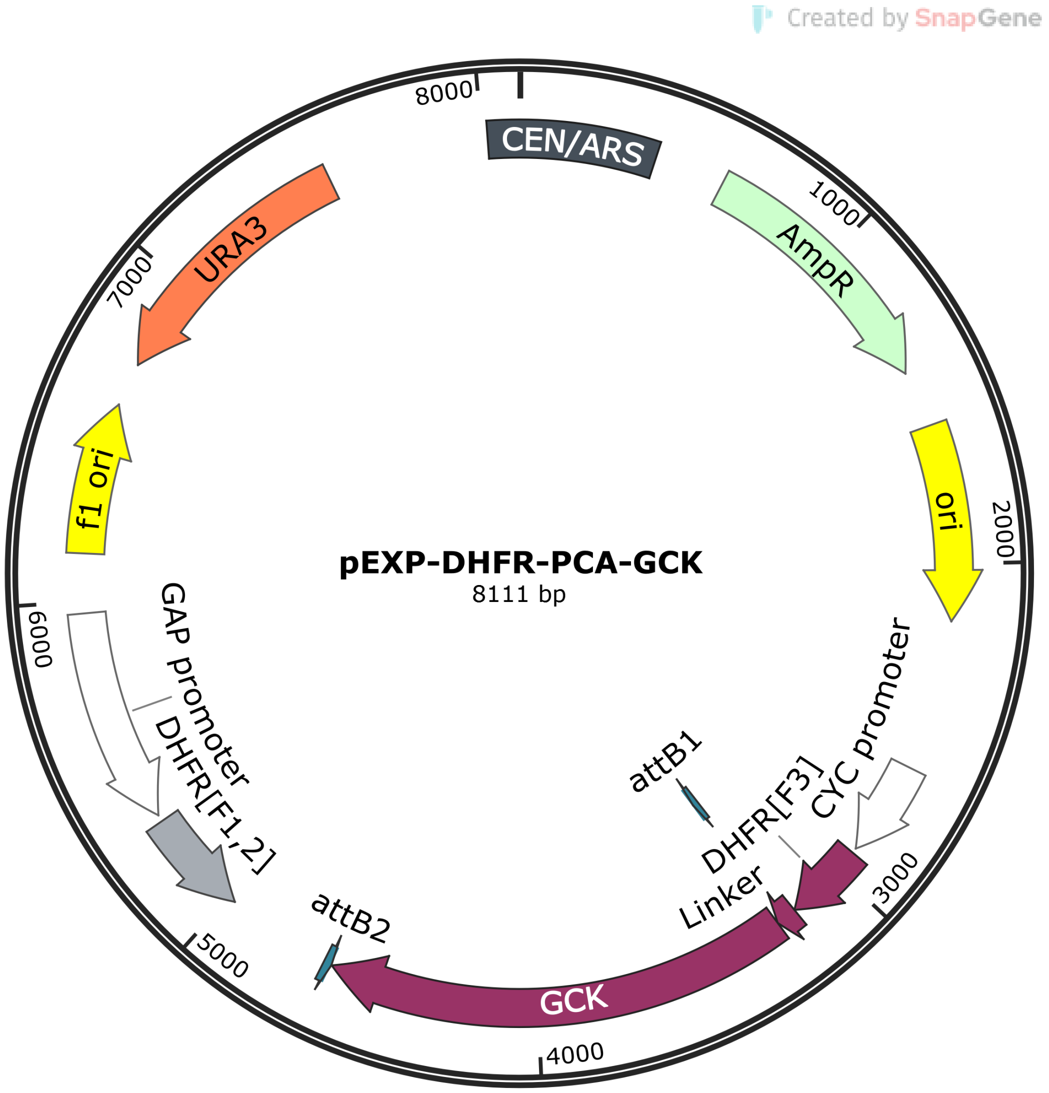

Figure S18. Plasmid map of pEXP-DHFR-PCA-GCK. A depiction of the DHFR-PCA expression system. Note that GCK is fused in the N-terminus to DHFR[F3]. The expression of DHFR[F3]-GCK is driven by the CYC promoter, while the expression of DHFR[F1,2] is driven by the GAP promoter. The plasmid contains part of an autonomously replicating sequence (ARS) and a centromere sequence (CEN) such that it is maintained at a low copy number in the cell. The plasmid map was generated using SnapGene® software (from Dotmatics; available at snapgene.com)

References

[1] Gersing S, Cagiada M, Gebbia M, Gjesing AP, Coté AG, Seesankar G, et al. A comprehensive map of human glucokinase variant activity. Genome Biology. 2023 Apr;24(1):97. Available from: https://genomebiology.biomedcentral.com/articles/10.1186/s13059-023-02935-8.

[2] Osbak KK, Colclough K, Saint-Martin C, Beer NL, Bellanné-Chantelot C, Ellard S, et al. Update on mutations in glucokinase (GCK), which cause maturity-onset diabetes of the young, permanent neonatal diabetes, and hyperinsulinemic hypoglycemia. Human Mutation. 2009 Nov;30(11):1512-26. Publisher: John Wiley & Sons, Ltd. Available from: https://onlinelibrary.wiley.com/doi/full/10.1002/humu.21110.

[3] Goddard TD, Huang CC, Meng EC, Pettersen EF, Couch GS, Morris JH, et al. UCSF ChimeraX: Meeting modern challenges in visualization and analysis. Protein Science: A Publication of the Protein Society. 2018 Jan;27(1):14-25.

[4] Pettersen EF, Goddard TD, Huang CC, Meng EC, Couch GS, Croll TI, et al. UCSF ChimeraX: Structure visualization for researchers, educators, and developers. Protein Science: A Publication of the Protein Society. 2021 Jan;30(1):70-82.

[5] Zhang J, Li C, Chen K, Zhu W, Shen X, Jiang H. Conformational transition pathway in the allosteric process of human glucokinase. Proceedings of the National Academy of Sciences. 2006 Sep;103(36):13368-73. Publisher: National Academy of Sciences. Available from: https://www.pnas.org/content/103/36/13368.
