# Supplementary material for: Characterizing glucokinase variant mechanisms using a multiplexed abundance assay
Source: Genome Biol. 2024 Apr 16;25:98. doi: 10.1186/s13059-024-03238-2 (PMC11021015; doi:10.1186/s13059-024-03238-2)
Supplement: Supplementary file 3 — Additional file 3. Review history. [file 13059_2024_3238_MOESM3_ESM.docx]

**1^st^ round**

**Reviewer 1**

This paper presents a comprehensive study investigating the impact of amino acid substitutions on the abundance of human glucokinase (GCK), complementing their recent study of GCK activity published in Genome Biology. It is very nice to see two complementary assays performed for the same system, as that is what allows us to obtain real mechanistic insight. The paper was nice to read - very clearly written with a highly detailed materials and methods explaining each step of the process. I appreciate that the authors have made sequencing data as well as abundance measurements freely available. I am supportive of publication in Genome Biology, but I have a few comments for the authors to consider.


-43% of loss-of-activity variants being due to reduced abundance is a bit lower than I would have intuitively guessed - I would have predicted most of them to be due to a loss of stability. How does that compare to what would be predicted to be destabilized using Rosetta ΔΔG? I slightly worry that this lack of correspondence between experiments could be reflecting experimental noise in one or both. It certainly would have been nice to have an internal replicate, although I'm not suggesting it would be necessary to do at this point. One thing that would somewhat reassure me is that the abundance measurements should show stronger correlation with the ΔΔG measurements, while the activity measurements, being independent of mechanism, should show stronger correlation with an evolution based metric (e.g. the authors could use their own ΔΔE score, or just download EVE scores).

-To what extent could the differences between big and small domains be explained by the fact that the big domain has a greater proportion of buried residues, and the small domain a greater proportion of surface residues? If you control for buried vs surface, or ΔΔG, is there still a difference?

-Is it possible to calculate ΔΔG values based on the effect on glucose or analogue binding to get a better idea of mutations acting directly via disruption of ligand binding, using a ligand-bound structure? I don't know if Rosetta can do it, but there are some tools like mCSM-lig.

-Instead of a barcoding strategy, the authors are using a direct tile sequencing approach. Have they considered the (high) likelihood of generating PCR errors that could introduce biases in the data.

-The abundance scores are an important part of this study, and could do with being better explained in the main text. I assume it is something like 1 is defined to be the mean of wild-type/synonymous, and 0 the mean of the nonsense variants, but it would be helpful to clarify this for the reader early on.

-It wasn't obvious to me which terminus of GCK is fused to DHFR

**Reviewer 2**

This is a report of a systematic study of all possible amino acid substitutions in GCK, to distinguish between loss of function variants that affect function vs. stability and abundance of the protein. A multiplex DHFR-PCA assay in yeast uses survival as a proxy for variant abundance with RNAseq as the readout. The results are compared to functional evaluation of all GCK variants previously published by the authors to catalogue the mechanism for each variant and make structural inferences about the protein. The results are convincing, novel and interesting. Some improvements to the manuscript are suggested.

1. My only validity concern is whether convincing evidence can be presented in a revised version, to show that structural alterations in the GCK part of the hybrid with the DHFR fragment do not affect the interaction of the fragment with its partner. Can a 3D molecular model be shown?
2. The Conclusion session is much too long and repeats at length what has already been discussed under Results and Discussion. It will be more pithy and effective if reduced to less than one-third of its current length.
3. Lines 149-150: The sentence "Potentially, some of these variants might be less active at 37 °C, the temperature at which abundance was assayed" appears to attempt to explain why low-abundance variants have normal or even increased function; it does not. Please rephrase and clarify.
4. Lines 76-83: it is customary to briefly (one sentence or, at most, two) pre-announce the results of the study at the very end of the Introduction section. This is not the place for longer explanations.
5. Line 147: loose vs. lose

**Reviewer 3**

The authors have used a previously described deep mutational scanning library coupled to a reporter assay to study the relation between GCK activity and abundance, where they find that almost 50% of hypoactive variants are characterized by low abundance and they also identified variants that affect protein stability and conformational dynamics.
Major points:
1. Perhaps I missed it, but I could not find a precise definition of the activity scores used either in this manuscript or in the recent publication by the same authors (Gersing et al. Genome Biology (2023) 24:97). This should be clearly described in the Materials and Methods Section
2. While the approach and conclusions in the present manuscript appear sound, there is relatively limited new information over what has already been published in the above reference and what is generally known about mutational effects on proteins. Mainly, the authors have used a DHFR protein complementation assay to estimate relative protein levels and to demonstrate, unsurprisingly that mutations in the large domain at buried sites, decrease protein levels in vivo. Mutations at a disordered stretch in the inactive (super-open) conformation in the small domain that becomes buried in the active conformation also decrease activity, by increasing the energy of the closed conformation. This is also not surprising as the super-open conformation is highly populated in the absence of substrate and mutations at disordered residues are unlikely to alter stability or in vivo levels of expressed protein. Another region around helix 13 was also found to be mutationally sensitive, consistent with an earlier study of Larion and Miller in 2009.

So in summary while this is a sound study with clear conclusions, it does not represent a significant conceptual advance or provide a large body of novel, disease relevant data.


Minor points:
1. Figure 1B: there are multiple lanes of cells spotting but no dilutions or DMSO/MTX concentrations are indicated.
2. Adding a schematic for the construct design would be useful, to show the relative positions of the DHFR fragment relative to the rest of the coding region.
3. Figure 2A: It would useful to provide domain information and residue burial (ASA) information along with the the heat map using appropriate colored bar above the heat map.
4. Are any of the assayed mutations with decreased activity due to decreased abundance prevalent in human disease associated mutations? If so, what is their frequency relative to mutations that directly affect specific activity?

**Authors’ response**

Reviewer #1: This paper presents a comprehensive study investigating the impact of amino acid substitutions on the abundance of human glucokinase (GCK), complementing their recent study of GCK activity published in Genome Biology. It is very nice to see two complementary assays performed for the same system, as that is what allows us to obtain real mechanistic insight. The paper was nice to read - very clearly written with a highly detailed materials and methods explaining each step of the process. I appreciate that the authors have made sequencing data as well as abundance measurements freely available. I am supportive of publication in Genome Biology, but I have a few comments for the authors to consider.

Our response:

We thank the reviewer for the kind words on our work and for spending time to provide valuable feedback.

-43% of loss-of-activity variants being due to reduced abundance is a bit lower than I would have intuitively guessed - I would have predicted most of them to be due to a loss of stability. How does that compare to what would be predicted to be destabilized using Rosetta ΔΔG? I slightly worry that this lack of correspondence between experiments could be reflecting experimental noise in one or both. It certainly would have been nice to have an internal replicate, although I'm not suggesting it would be necessary to do at this point. One thing that would somewhat reassure me is that the abundance measurements should show stronger correlation with the ΔΔG measurements, while the activity measurements, being independent of mechanism, should show stronger correlation with an evolution based metric (e.g. the authors could use their own ΔΔE score, or just download EVE scores).

Our response:

We agree that 43% is a bit lower than what we found previously (50%-60%, PMID: 33779753). As suggested, we have now included the fraction of hypoactive variants predicted to be destabilized using Rosetta ΔΔGs (43% using the super-open structure), which is identical to the percentage found using experimental scores (43%) (l. 162- 166). An explanation for the slightly lower percentage than expected could be the conformational flexibility of the small domain, which appears to be mutationally tolerant with respect to abundance. In addition, to address this issue, we assessed the correlations between activity/abundance scores and ΔΔG/GEMME scores (a sequence/evolution-based metric). We found that abundance scores show stronger correlation with ΔΔG values, while activity scores show stronger correlation with GEMME scores (l. 156-159, Fig. S5, S6, S7 and S8).

-To what extent could the differences between big and small domains be explained by the fact that the big domain has a greater proportion of buried residues, and the small domain a greater proportion of surface residues? If you control for buried vs surface, or ΔΔG, is there still a difference?

Our response:

We thank reviewer 1 for pointing out this important control. We have now included the percentages of low-abundance residues in each domain that have been adjusted for the number of buried residues in each domain (l. 146-149). Indeed, we still observe a difference with ratios of 57%/31% and 55%/17% (large domain/small domain) when using the super-open and closed structures, respectively, for normalization.

-Is it possible to calculate ΔΔG values based on the effect on glucose or analogue binding to get a better idea of mutations acting directly via disruption of ligand binding, using a ligand-bound structure? I don't know if Rosetta can do it, but there are some tools like mCSM-lig.

Our response:

This is a great question. We have now repeated ΔΔG predictions with and without glucose bound to identify variants predicted to decrease glucose binding affinity (l. 169- 171, Fig. S10)

-Instead of a barcoding strategy, the authors are using a direct tile sequencing approach. Have they considered the (high) likelihood of generating PCR errors that could introduce biases in the data.

Our response:

This is a good point. We have now addressed this concern in the Methods and Materials section (l. 422-428).

-The abundance scores are an important part of this study, and could do with being better explained in the main text. I assume it is something like 1 is defined to be the mean of wild-type/synonymous, and 0 the mean of the nonsense variants, but it would be helpful to clarify this for the reader early on.

Our response:

We completely agree. The scores are now explained in the main text (l. 124-126).

-It wasn't obvious to me which terminus of GCK is fused to DHFR

Our response:

We have clarified that the N-terminus of GCK was fused to DHFR (l. 310-312, Fig. S18).

Reviewer #2: This is a report of a systematic study of all possible amino acid substitutions in GCK, to distinguish between loss of function variants that affect function vs. stability and abundance of the protein. A multiplex DHFR-PCA assay in yeast uses survival as a proxy for variant abundance with RNAseq as the readout. The results are compared to functional evaluation of all GCK variants previously published by the authors to catalogue the mechanism for each variant and make structural inferences about the protein. The results are convincing, novel and interesting. Some improvements to the manuscript are suggested.

Our response:

We thank the reviewer for the encouraging words and the suggestions to improve our manuscript.

1.My only validity concern is whether convincing evidence can be presented in a revised version, to show that structural alterations in the GCK part of the hybrid with the DHFR fragment do not affect the interaction of the fragment with its partner. Can a 3D molecular model be shown?

Our response:

Although we cannot completely rule out that mutations in GCK affect the interaction of the two DHFR fragments, we show that abundance scores correlate with GCK protein levels when expressed without the DHFR fragment (Fig. S1BC), suggesting that alterations in GCK protein abundance is the main cause for altered growth in the assay. In addition, a previous study validated, using ~2000 yeast proteins, that yeast growth in the DHFR-PCA is proportional to the abundance of the DHFR[F3] fusion protein (PMID: 24813894). Furthermore, to avoid aberrant interactions between GCK and the DHFR fragments, DHFR[F3] and GCK were separated by a linker of 21 residues. The presence of a linker has now been clarified (l. 309-310, Fig. S18).

2.The Conclusion session is much too long and repeats at length what has already been discussed under Results and Discussion. It will be more pithy and effective if reduced to less than one-third of its current length.

Our response:

We agree and have reduced the repetition in the Conclusion section (l. 278-294).

3.Lines 149-150: The sentence "Potentially, some of these variants might be less active at 37 °C, the temperature at which abundance was assayed" appears to attempt to explain why low-abundance variants have normal or even increased function; it does not. Please rephrase and clarify.

Our response:

We apologize for the vague explanation which we have clarified (l. 173-178).

4.Lines 76-83: it is customary to briefly (one sentence or, at most, two) pre-announce the results of the study at the very end of the Introduction section. This is not the place for longer explanations.

Our response:

The results are now only mentioned briefly (l. 87-92).

5.Line 147: loose vs. lose

Our response:

This mistake has now been corrected (l. 170).

Reviewer #3: The authors have used a previously described deep mutational scanning library coupled to a reporter assay to study the relation between GCK activity and abundance, where they find that almost 50% of hypoactive variants are characterized by low abundance and they also identified variants that affect protein stability and conformational dynamics.

Our response:

We thank the reviewer for spending time to provide valuable feedback on our work.

Major points:

1.Perhaps I missed it, but I could not find a precise definition of the activity scores used either in this manuscript or in the recent publication by the same authors (Gersing et al. Genome Biology (2023) 24:97). This should be clearly described in the Materials and Methods Section

Our response:

We apologize for this and have now included a description of the activity scores, albeit in the Results and Discussion section (l. 155-156).

2.While the approach and conclusions in the present manuscript appear sound, there is relatively limited new information over what has already been published in the above reference and what is generally known about mutational effects on proteins. Mainly, the authors have used a DHFR protein complementation assay to estimate relative protein levels and to demonstrate, unsurprisingly that mutations in the large domain at buried sites, decrease protein levels in vivo. Mutations at a disordered stretch in the inactive (super-open) conformation in the small domain that becomes buried in the active conformation also decrease activity, by increasing the energy of the closed conformation. This is also not surprising as the super-open conformation is highly populated in the absence of substrate and mutations at disordered residues are unlikely to alter stability or in vivo levels of expressed protein. Another region around helix 13 was also found to be mutationally sensitive, consistent with an earlier study of Larion and Miller in 2009. So in summary while this is a sound study with clear conclusions, it does not represent a significant conceptual advance or provide a large body of novel, disease relevant data.

Overall we agree that many of our observations are in line with expected consequences of protein biophysics, like the destabilizing effect of mutations in buried residues in the large domain, or conceptually that mutations can shift the populations of open/closed states. The strength of our work is that it quantifies these aspects for nearly all variants. Our study provides abundance scores for 8822 GCK variants – a major advancement compared to the previously known stabilities of GCK variants. For example, the finding that many variants in the small domain affect abundance relatively little was surprising. Accordingly, these data, combined with our previous work, provide information on the loss of function mechanism for nearly all GCK variants. In addition, we provide extensive computational and experimental data on the impact of GCK residues on protein stability and dynamics, that are in line with prior studies focusing on limited regions or variants.

Minor points:

1.Figure 1B: there are multiple lanes of cells spotting but no dilutions or DMSO/MTX concentrations are indicated.

Our response:

We apologize for this omission and have added the details to Fig. 1B and Fig. 4.

2.Adding a schematic for the construct design would be useful, to show the relative positions of the DHFR fragment relative to the rest of the coding region.

Our response:

This is a good idea and we have added a plasmid map as a supplementary figure (Fig. S18)

3.Figure 2A: It would useful to provide domain information and residue burial (ASA) information along with the the heat map using appropriate colored bar above the heat map.

Our response:

We agree and have added domain information and the relative solvent accessible surface area (rASA) to Fig. 2A.

4.Are any of the assayed mutations with decreased activity due to decreased abundance prevalent in human disease associated mutations? If so, what is their frequency relative to mutations that directly affect specific activity?

Our response:

This is a great question. We have now added data on the activity and abundance scores of 57 GCK-MODY variants and find that 41% of the hypoactive disease- associated mutations also show low abundance, which is comparable to the 43% observed for all variants (l. 166-168, Fig. S9).

**2^nd^ round**

**Reviewer 1**

I am happy with the authors' response to my queries. I just have one tiny point remaining. "Low-abundance variants were still enriched in the large domain when normalizing for the number of buried residues in the two domains, with ratios of 57%/31% and 55%/17%". I'm not sure what "normalizing" means here. Are these percentages only considering buried residues?

**Reviewer 3**

The authors have satisfactorily addressed most of my concerns in the revised manuscript.

**Authors’ response**

we have addressed Reviewer 1’s single point, and have changed the sentence

to: “The ratios of low abundance residues to buried residues were 57% (55%) and 31%

(17%) for the large and small domain, respectively, when evaluated on the super-open

(closed) structure. Thus, low abundance variants were still enriched in the large

domain when the difference in the number of buried residues in the two domains was

taken into account.”
